# The Regulation of Metabolic Homeostasis by Incretins and the Metabolic Hormones Produced by Pancreatic Islets

**DOI:** 10.2147/DMSO.S415934

**Published:** 2024-06-13

**Authors:** Joshua Reed, Stephen C Bain, Venkateswarlu Kanamarlapudi

**Affiliations:** 1 Institute of Life Science, Medical School, Swansea University, Swansea, SA2 8PP, UK

**Keywords:** type 2 diabetes, obesity, insulin, glucagon, GLP-1, GLP-1R, incretin, metabolism

## Abstract

In healthy humans, the complex biochemical interplay between organs maintains metabolic homeostasis and pathological alterations in this process result in impaired metabolic homeostasis, causing metabolic diseases such as diabetes and obesity, which are major global healthcare burdens. The great advancements made during the last century in understanding both metabolic disease phenotypes and the regulation of metabolic homeostasis in healthy individuals have yielded new therapeutic options for diseases like type 2 diabetes (T2D). However, it is unlikely that highly desirable more efficacious treatments will be developed for metabolic disorders until the complex systemic regulation of metabolic homeostasis becomes more intricately understood. Hormones produced by pancreatic islet beta-cells (insulin) and alpha-cells (glucagon) are pivotal for maintaining metabolic homeostasis; the activity of insulin and glucagon are reciprocally correlated to achieve strict control of glucose levels (normoglycaemia). Metabolic hormones produced by other pancreatic islet cells and incretins produced by the gut are also crucial for maintaining metabolic homeostasis. Recent studies highlighted the incomplete understanding of metabolic hormonal synergism and, therefore, further elucidation of this will likely lead to more efficacious treatments for diseases such as T2D. The objective of this review is to summarise the systemic actions of the incretins and the metabolic hormones produced by the pancreatic islets and their interactions with their respective receptors.

## Introduction

Maintaining metabolic homeostasis is essential in all living organisms, as it provides energy in the form of adenosine trisphosphate (ATP) required for cellular processes to proceed.^1^,^2^ In healthy humans, the complex biochemical interplay between organs maintains metabolic homeostasis.^3^,^4^ Disorders that affect this biochemical interplay, such as diabetes and obesity, result in impaired metabolic homeostasis in individuals.^4^,^5^ The main hormones that regulate metabolic homeostasis are insulin and glucagon, which are secreted into circulation as required by islet-beta and alpha cells, respectively.^6^,^7^ Insulin is considered to be an anabolic hormone due to its ability to positively influence glycogen, protein, fatty acid and triacylglycerol synthesis in tissues, and glucagon is considered to be a catabolic hormone due to its ability to promote the breakdown of the same in tissues.^4^,^8^ The activity of both insulin and glucagon, which play a vital role in maintaining metabolic homeostasis, is required to accomplish strict control of glucose levels (normoglycaemia).^6^,^7^,^9^ It has also been elucidated that other hormones produced by other pancreatic islet cells and the gut are crucial for maintaining metabolic homeostasis, in addition to insulin and glucagon.^10–12^ Glucose when administered orally promotes significantly higher pancreatic insulin secretion than when it is administered intravenously, which is termed the “incretin effect”.^11^,^13^ This led to the identification of two gut hormones that produce the incretin effect: glucagon-like peptide-1 (GLP-1) and gastric inhibitory peptide (GIP) which are produced by L and K cells of the gut, respectively.^14^,^15^ The incretin hormones GLP-1 and GIP are confirmed to be crucial for maintaining metabolic homeostasis via augmentation of insulin secretion from islet beta-cells. The incretin effect is responsible for 60–70% of insulin secretion after glucose consumption in healthy individuals.^16^,^17^ Thus, the activity of the pancreatic islets and the L and K cells are closely coupled to maintain metabolic homeostasis in humans.

Obesity and type 2 diabetes (T2D) are the main metabolic disorders given their global prevalence and economic burden.^18^ Globally, approximately (∼) 382 million patients were estimated to have diabetes in 2013 and ∼600 million individuals were classified as obese (BMI≥30 kg/m^2^) in 2014 and these numbers will increase in the future.^18^,^19^ Inadequate energy spending together with excessive energy intake and storage induces obesity, which causes gain of weight.^18^ Whilst obesity manifests due to excessive diets and sedentary lifestyles, it has been established that this condition also has a genetic aetiology in some individuals, usually involving the genes involved with the central nervous system (CNS) regulation of hunger and satiety, particularly the hypothalamic leptin-melanocortin pathway.^20^ Mutations in the melanocortin-4 receptor gene account for the most common form of monogenetic obesity.^21^ Lifestyle changes, in the form of a healthier diet and increased exercise to promote weight loss are the first treatment option for obesity, but this is often not an effective long-term strategy for patients.^18^,^22^ Pharmacological intervention is therefore usually required to assist in long-term weight loss in obese patients, and there several available drugs for this but they often exhibit insufficient efficacy and dubious safety.^22^,^23^ Bariatric surgery is another option for inducing weight loss in obese individuals if the aforementioned treatment strategies fail, although patients often develop post-surgery complications requiring further therapeutic intervention.^18^,^24^

The incidence of T2D continues to increase and by 2035 it is estimated that there will be >590 million patients diagnosed with this condition.^19^,^25^ Diabetes is defined by the World Health Organisation as a metabolic disorder of multiple aetiology characterised by chronic hyperglycaemia with disturbance of carbohydrate, fat, and protein metabolism resulting from defects in insulin secretion, insulin action, or both.^26^ Since ~90% of diabetic patients have T2D, it is the most common form of diabetes.^25^ The bulk of the remaining 10% of the patients are diagnosed with type 1 diabetes (T1D), although other types exist that are rare.^27^ Defects in insulin secretion from pancreatic beta-cells and insulin resistance in peripheral tissue result in T2D.^28^,^29^ The aetiology of T2D has not been firmly established but diets involving excessive nutrient consumption are thought to be key to the development of this disease. Approximately 90% of patients are obese or overweight when they are diagnosed to have T2D.^30^ The impaired metabolic homeostasis caused by diabetes results in hyperglycaemia. There are several pharmacological treatments available to augment insulin secretion or reduce peripheral tissue insulin resistance in addition to patients adopting a healthier diet and increased exercise to achieve weight loss, which promotes normoglycaemia and alleviates the disease phenotype.^31^ Bariatric surgery is another option for obese T2D patients should lifestyle changes and pharmacological treatments not produce sufficient results, although as aforementioned this surgery is typically associated with post-surgery complications in patients.^18^ Although treatments are available for diabetes and obesity, there are multiple long-term complications: they remain the leading causes of cardiovascular disease, eye pathology, lower limb amputation and end-stage renal disease.^26^,^32^ Additionally, treatments are often associated with side effects and/or exhibit insufficient efficacy.^18^,^22–24^ Therefore, more efficacious treatments for diabetes and obesity are highly desirable.

The objective of this review is to discuss the established and speculative roles that hormones released by the pancreatic islets (such as insulin and glucagon) and digestive system (incretins [GLP-1 and GIP]) play in regulating metabolic homeostasis in healthy individuals. Additionally, this review aims to highlight the currently incompletely understood complex synergistic interactions between these different hormones involved in regulating metabolic homeostasis. Some of the other hormones involved in metabolic homeostasis are also briefly mentioned in this review. Further understanding the systemic actions of each hormone in isolation or combination with others and their interactions with their respective receptors will likely enable a better understanding of the processes by which humans maintain metabolic homeostasis under healthy conditions and how these are altered during disease, which will then likely yield more effective new therapeutic options for metabolic diseases such as diabetes and obesity. Whilst great advancements have been made over the last century with regard to understanding the regulation of metabolic homeostasis which has yielded new therapeutic options for diseases such as T2D, it is unlikely that highly desirable more efficacious treatments will be developed for metabolic disorders until the complex systemic regulation of metabolic homeostasis becomes more intricately understood.

### Hormones Produced by the Pancreatic Islets

#### Insulin and Glucagon Overview

The main hormones that regulate metabolism are insulin and glucagon, which are produced by pancreatic beta- and alpha-cells, respectively.^10^,^33^ Maintaining normoglycaemia is a complex process regulated by the coordinated secretion of glucagon and insulin.^9^ Insulin is regarded as the metabolic anabolic hormone given its effects on target tissues, whereas glucagon is regarded as the metabolic catabolic hormone.^7^,^8^ The release of glucagon and insulin in both cell types is coupled to the intracellular ATP/ADP ratio, which in turn is determined by the levels of glucose and other nutrients in the bloodstream.^5^,^34^ A raised ATP/ADP ratio in beta-cells results in insulin secretion, whereas in alpha-cells an increased ATP/ADP ratio prevents glucagon secretion.^34^,^35^ Evolution has developed pancreatic alpha and beta-cells to act as the ‘master regulators’ of metabolism, as their ability to secrete key hormones involved in promoting metabolic homeostasis is determined by nutrient levels in circulation.

Blood glucose concentration needs to be maintained within a narrow range as hypoglycaemia and hyperglycaemia are associated with pathology.^3^,^12^ The overall purpose of the activity of these hormones is to ensure that all biological processes throughout the body are provided with adequate energy in the form of ATP for them to occur, by promoting anabolism (in the case of insulin) and catabolism (in the case of glucagon) of carbohydrates and fats in tissues as required.^7^,^36^ The pancreas is the key organ for promoting metabolic homeostasis.^9^,^37^ It is estimated that 4–5% of the pancreas is comprised of endocrine cells (which secrete insulin and glucagon), which are found in small clusters scattered throughout the pancreas called the islets of Langerhans, and the rest of this organ is comprised of exocrine tissue which is involved in digestion.^38^ In a healthy human, ~70% of the cells in a pancreatic islet of Langerhans, are beta-cells, 20% are alpha-cells, and the remaining 10% consist of (in order of most to least prevalent) delta, gamma and epsilon cells.^10^ The different cell types in the islets are known to be able to influence each other’s activity via paracrine communication, and additionally, they can regulate their activity via autocrine feedback.^37^ Disruption of hormone production and secretion from the pancreatic islets, as well as impaired action on target tissues, results in impaired metabolic homeostasis and diseases such as diabetes.^5^

#### Insulin

The human insulin gene is located on chromosome 11p15.5 and consists of three exons and two introns.^39^ Pancreatic beta-cells are responsible for the synthesis and secretion of insulin; the insulin gene is only transcribed to mRNA in these cells.^5^,^40^ Pancreatic beta-cells found in the islets of Langerhans are designed to act as ‘fuel sensors’ and produce and secrete insulin in response to the presence of adequate levels of nutrients in circulation.^41^,^42^ Each mouse pancreatic beta-cell contains ~13,000 insulin granules, which account for >10% of the total cell volume, and each granule contains ~200,000 insulin molecules.^5^ In islet beta-cells, the insulin gene encodes preproinsulin, which is a 110-amino acid (aa) insulin precursor.^5^,^43^ Preproinsulin undergoes intracellular processing to form proinsulin, which is further processed resulting in the insulin that is secreted into circulation. Insulin consists of 51aa with a molecular weight of 5.8 kDa.^5^ The fusion of insulin granules with the plasma membrane results in insulin secretion through exocytosis of granule content.^35^ Under normal circumstances, islet beta-cell morphology is characterised by a large and stable number of intracellular mature insulin vesicles, which is sustained through the balance between biosynthesis, degradation and secretion.^44^ During starvation, beta-cells adapt by markedly reducing their number of intracellular insulin vesicles via degradation, and these cells can rapidly replenish their insulin stores and return to a normal morphology in response to refeeding.^44^,^45^ In response to overnutrition, obesity and insulin resistance, beta-cells adapt by increasing their mass and/or number, both of which result in increased capacity of insulin secretion.^46^ The metabolism of islet beta-cells is designed to be sensitive to blood glucose levels as the hexokinase isozyme found in these cells is glucokinase which has a reduced affinity for substrate, resulting in glucose catabolism occurring when glucose concentration in the blood is 5mM or higher.^41^,^47^ From a bioenergetics point of view, islet beta-cells are unique, as their intracellular ATP/ADP ratio is regulated by ATP supply and not ATP demand like in most other cell types.^41^,^42^

Studies have unravelled that proton leak is unusually high in pancreatic beta-cells in comparison to other cell types such as muscle cells.^41^,^48^,^49^ It has been reported that up to 75% of their metabolism is unproductive due to proton leak (premature leakage of protons through the inner mitochondrial membrane not mediated by ATP synthase) which is staggering, given that insulin secretion occurs in an ATP-dependent manner.^41^ Even after years of research, it is still largely unknown why these cells have such inefficient metabolism.^41^,^48^,^50^ Reactive oxygen species (ROS) (which are generally thought to be cytotoxic molecules) produced during metabolism have been shown to amplify insulin secretion.^42^ Interestingly, islet beta-cells express reduced levels of antioxidant enzymes to deal with ROS compared to other cell types,^51^,^52^ indicating that ROS must be useful to these cells.^41^ However, it is also known that an excess of ROS in these cells leads to cell damage and decreased viability.^50^ Generally speaking, the amount of postprandial insulin released into circulation is directly proportional to the levels of nutrients ingested. This is due to these cells being designed to have ATP levels that reflect the nutrient levels in circulation.^53–55^

In the established model of insulin secretion, islet beta-cells’ glucokinase firstly “senses” that the serum concentration of glucose is 5mM or greater, which triggers glycolysis leading to ATP production, glucose oxidation and ultimately insulin secretion; hence, glucose metabolism and the subsequent sequence of events which lead to insulin secretion are known as glucose-stimulated insulin secretion (GSIS).^47^,^53^,^56^ GSIS is a biphasic event in healthy humans: first phase insulin release lasts only a few minutes whilst the prolonged second phase sustains insulin secretion to deal with post-prandial nutrient loads.^5^,^55^ During the first phase of insulin secretion, islet beta-cells readily secrete synthesised insulin stored in vesicles during the initial minutes after these cells “sense” blood glucose concentrations of ≥5mM.^53^,^55^ In humans, the first-phase insulin secretion peaks at 1.4 nmol/min, whereas the second phase results in insulin secretion at a rate of ~0.4 nmol/min.^5^ The second phase of insulin secretion consists of a prolonged response, where islet beta-cells synthesise and secrete insulin.^5^,^53^,^55^ Hence, this phase mediates a steady secretion of insulin which results in a lower insulin level in the blood compared to the first phase.^53^,^55^ It is known that both phases of insulin secretion are ATP-dependent processes.^57^,^58^

**Figure 1:**
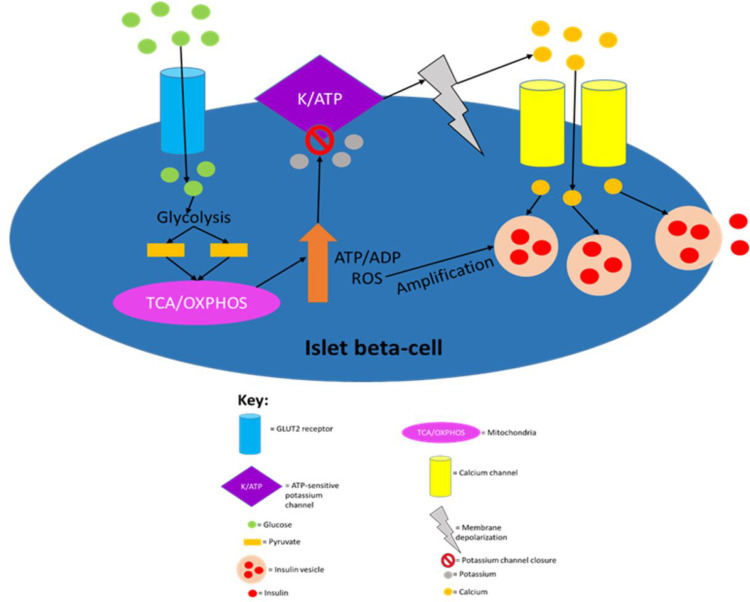
The canonical model of insulin secretion in pancreatic beta-cells. When the plasma glucose concentration rises to 5mM or higher, glucose transporter 2 (GLUT2), an insulin-insensitive glucose transporter, allows glucose to enter islet beta-cells from the circulation. The product of glycolysis is pyruvate which increases tricarboxylic acid cycle turnover and oxidative phosphorylation. This results in a rise in the cell’s ATP/ADP ratio which leads to the closure of ATP-sensitive potassium channels, causing membrane depolarisation (due to potassium becoming sequestered in the cell) and subsequent calcium influx. The resulting calcium influx then triggers insulin secretion by causing insulin vesicles to fuse with the plasma membrane. ROS produced during oxidative phosphorylation have been shown to amplify insulin secretion, although it is not understood how they do so mechanistically. This figure and the information in its legend are adapted from these studies.^41^,^48^,^53^,^55^

Glycolytic products activate the tricarboxylic acid cycle in the mitochondria which leads to electron transport across the electron transport chain resulting in the production of a protonmotive force and subsequent ATP production via ATP synthase activity.^4^ The ATP production induces first-phase insulin secretion as the now raised ATP/ADP ratio in the cell leads to the closure of ATP-sensitive potassium channels, which then results in membrane depolarisation.^41^ The membrane depolarisation causes voltage-gated calcium channels in the cell membrane to open leading to calcium ion influx into the cell, which then results in the exocytosis of insulin vesicles stored in the cytoplasm.^41^,^48^ The ATP production also allows for second-phase insulin secretion, partly because both phases are similar mechanistically as both require ATP for the closure of ATP-sensitive potassium channels.^41^,^53^ However, they are also distinct as successful second-phase insulin secretion requires synthesizing insulin and insulin vesicles, which are not thermodynamically favourable processes; hence, ATP hydrolysis will be required in order to provide the energy for these processes to occur.^4^,^53^,^55^ It has recently been elucidated that fatty acids not only augment GSIS but also induce insulin secretion in the absence of glucose.^59^ However, fatty acid-induced insulin secretion in the absence of glucose is ~40% that of GSIS. Figure 1 summarises the established model of insulin secretion.

The nervous system also regulates insulin secretion from islet beta-cells.^40^ Vagal nerve stimulation induces insulin secretion during the fed state but not the fasting state.^60^ When food is smelled, seen or enters the gastrointestinal tract (GIT), islet cell cholinergic muscarinic receptor activation induces insulin secretion.^61^ The vagal nerve stimulation of insulin release is postulated to facilitate the “cephalic phase” of insulin secretion, which occurs even before blood glucose or fat levels rise.^60^ A recent systematic review found that there is little evidence for a physiologically relevant cephalic phase insulin response, although more than half of the studies examined documented its presence.^62^ Therefore, the existence of the cephalic phase of insulin secretion remains controversial and it appears to have minimal, if any, physiological relevance. The parasympathetic nervous system was also shown to potentiate insulin secretion during hyperglycaemic excursions in humans.^60^,^63^ Additionally, the nervous system has negative regulatory effects on insulin secretion, as catecholamines, through alpha2-adrenoreceptors, are known to usually inhibit insulin release during exercise, by acting downstream on signalling pathways activated by nutrient secretagogues.^40^,^64^ Many of the mechanisms by which neurotransmitters regulate insulin secretion remain unclear and this area requires further investigation.^65^

The aa arginine promotes insulin secretion in an ATP-independent manner, as it increases potassium permeability resulting in depolarisation and subsequent insulin secretion.^40^,^66^ Leucine also positively regulates insulin secretion as it generates ATP via the Krebs cycle by allosterically stimulating glutamic dehydrogenase and producing alpha-ketoglutarate.^67^ How dietary amino acids modulate insulin secretion is discussed in more detail in another review.^68^ Proinsulin synthesis is induced in islet beta-cells when plasma glucose levels are greater than 2mM, ensuring that there is a sufficient reservoir of insulin secretory stores available for when secretion is needed to promote nutrient uptake from the circulation into tissues.^44^ Interestingly, other macronutrients (for example fatty acids) do not induce proinsulin biosynthesis, in spite of their ability to act as potent secretagogues for insulin secretion;^44^,^69^ it has been demonstrated that intracellular insulin stores can become depleted under chronic hyperlipidaemic conditions.^69^ Whilst some studies have found that neither exogenously added nor secreted insulin has any autocrine signalling effect, this remains an area of controversy.^70–72^

Once released into circulation, insulin mediates its anabolic effects on target tissues by binding to its receptor (insulin receptor) on target tissues.^40^ Insulin receptor is expressed in skeletal muscle, adipocytes, kidneys, brain, blood vessels, heart, liver, pancreatic endocrine cells (such as islet alpha and beta-cells), bone and the GIT.^8^,^40^ Glucose entry into these cells occurs in an ATP-independent manner and is mediated by glucose transporters (GLUTs).^73^ Three GLUT isoforms are expressed in skeletal muscle and adipose tissue: GLUT1, GLUT3 and GLUT4.^40^ The main mechanism by which insulin induces blood glucose uptake into skeletal muscle and adipose tissue is by stimulating the GLUT4 translocation from intracellular pools to the plasma membrane.^74^ The increased supply of glucose into these tissues results in the promotion of glycogen synthesis or increased fat storage under resting conditions.^8^,^74^ Insulin also increases the enzymatic activity of both hexokinase and 6-phosphofructokinase which results in enhanced glycolysis and glucose metabolism.^8^,^75^ Additionally, insulin also increases the activity of glycogen synthase and inhibits the activity of glycogen phosphorylase to further promote glycogen synthesis.^8^ Inhibition of glycogen phosphorylase is a very important effect of insulin as the catalytic capacity of this enzyme is 50-fold higher than glycogen synthase in human skeletal muscle.^8^ Subsequently, the promotion of net intracellular glycogen synthesis requires glycogen phosphorylase activity to be inhibited by ~99%.

One study showed that insulin’s ability to induce glycogen synthesis was dependent on this hormone’s ability to inhibit phosphorylase activity - the stimulation of glucose uptake and glycogen synthase was not enough to promote net glycogen synthesis.^76^ Skeletal muscle accounts for 60–70% of the blood glucose uptake due to insulin action, and it has been estimated that adipose tissue accounts for ~10% of the insulin-stimulated whole-body glucose uptake.^40^ Insulin also promotes protein synthesis in skeletal muscle, liver, adipose tissue and other tissues, whilst simultaneously decreasing the rate of protein degradation in these tissues.^8^,^40^ The liver, similar to islet beta-cells, does not depend on insulin to uptake glucose from circulation.^40^ When blood glucose concentrations are high enough, liver cells uptake glucose from circulation and store it as glycogen.^4^,^40^ Insulin stimulation results in fatty acid synthesis in the adipose tissue, liver and lactating mammary glands, whilst simultaneously inhibiting lipolysis and fatty acid oxidation in the liver, skeletal muscle and adipose tissue.^8^,^40^ Insulin receptor activation in islet alpha-cells suppresses glucagon secretion, and interestingly, pancreatic beta-cells also express insulin receptor and it has been postulated that insulin may positively regulate GSIS and promote cellular proliferation.^40^,^77^,^78^ However, a recent study demonstrated in mice that secreted insulin in response to GSIS mediates insulinostatic actions via insulin receptor on islet beta-cells.^79^ Insulin has also been shown to have anabolic effects on bone by stimulating osteoblasts whilst suppressing osteoclast function.^80^ Given the effects of insulin on metabolic fuel storage, it is considered to be an anabolic hormone.^8^

Although the brain is not dependent on insulin for glucose uptake, insulin receptors have been detected in the hypothalamus, olfactory bulb, retina, hippocampus, vessels of the choroid plexus and regions of the striatum and cerebral cortex.^81^ It has been hypothesised that insulin moves through the blood-brain barrier into the brain and acts as a neuropeptide positively regulating satiety, olfaction, cognition and memory as well as regulating systemic functions such as hepatic glucose production.^82^,^83^ However, it has not been elucidated if insulin is locally synthesised in the brain or if it is transported from circulation in order to mediate any effects.^40^,^84^ Additionally, by increasing lipid storage in adipose tissue, insulin indirectly induces leptin release from adipocytes, which strongly mediates satiety by acting on hypothalamic brain cells expressing the leptin receptor.^8^,^85^ The importance of leptin is demonstrated in leptin-deficient humans and mice, which develop obesity and have chronic hunger.^86^

Plasma adiponectin levels are also regulated by insulin action as the increased adiposity induced by the anabolic effects of insulin reduces adiponectin secretion from adipocytes: circulating adiponectin levels are inversely correlated with adiposity.^87^ It has been shown that adiponectin increases insulin sensitivity in the liver and skeletal muscle, which is thought to be due to its ability to increase lipid and glucose metabolism.^88^ Hence, the effect of insulin counteracts the action of adiponectin by promoting nutrient storage in these tissues; the synergistic action of both of these hormones may, therefore, produce a desirable net effect on nutrient anabolism/catabolism. Interestingly, adiponectin inhibits lipolysis in adipocytes giving it insulin-like effects on adipocytes, so during fasting and a subsequent decrease in adiposity, the actions of this hormone prevent lipolysis.^89^ This seems counterproductive as during the fasting state adipocytes need to release stored fat and it is likely glucagon action promotes net lipolysis in adipocytes- perhaps adiponectin reduces the rate of lipolysis to stop hyperlipidaemia. Overexpressing adiponectin in leptin-deficient (*ob/ob)* mice results in decreased plasma glucose and insulin levels, increased liver and muscle insulin sensitivity (which is thought to be due to decreased fat content), significantly increased mass of adipose tissue (these mice were morbidly obese) and a highly significant lowering of plasma triglycerides and FFAs. The diabetic phenotype in these mice was completely reversed by overexpressing adiponectin in these mice.^90^

Insulin was shown to reduce resistin mRNA levels in mouse 3T3-L1 adipocytes, suggesting that resistin may counteract the systemic anabolic effects of insulin and may therefore be important during fasting.^91^ Several studies have reported a positive relationship between insulin resistance, obesity and increased serum resistin in mouse models as increased resistin plasma levels were observed in diet-induced and *ob/ob* obese mice, anti-resistin antibodies increased insulin sensitivity in obese and insulin-resistant animals, healthy mice treated with recombinant resistin exhibited reduced glucose tolerance and insulin action, and resistin dampened insulin-induced glucose uptake in mouse adipocytes.^92^ It has been difficult to apply these findings to humans as resistin secretion is different between these two species. Resistin is mainly secreted from adipose tissue in mice whereas it is mainly secreted from monocytes in humans.^92^ Further, human resistin only shares 59% homology with mouse resistin at the amino acid level. Discrepancies exist in the data regarding the relationship between resistin and obesity and/or diabetes in humans as well as rodents.^92^ In human studies, resistin levels in circulation were either increased or unchanged in obese individuals with or without insulin resistance.^93^,^94^ Another study has shown that resistin expression was significantly increased in obese people but no association was found with T2D.^95^ In contrast, another study found that resistin levels were higher in T2D patients and individuals with impaired fasting glucose.^96^ Furthermore, some studies have observed that resistin is downregulated in the adipose tissue of obese mice.^97^ Therefore, it is not clear how resistin activity, as a result of insulin action, plays a role in obesity, insulin resistance and T2D given the findings from various studies.

Nitric oxide is a key molecule produced in the vasculature, which mediates endothelial-dependent relaxation and inhibits platelet aggregation, smooth muscle proliferation and cell adhesion.^40^ Insulin plays an important role in nitric oxide production and is therefore also important for maintaining cardiovascular homeostasis.^98^ The kidney expresses insulin receptors in the proximal tubules and insulin binding results in increased sodium reabsorption.^99^ Ghrelin is a hormone that increases appetite, gastric acid secretion and gastrointestinal motility.^100^,^101^ Ghrelin-secreting cells in the GIT express insulin receptor and insulin action negatively regulates ghrelin secretion.^102^ Hence, insulin indirectly reduces gastrointestinal motility, appetite and gastric acid secretion due to its suppression of ghrelin.

Knockout of insulin receptor in mice results in slight growth retardation at birth, but no metabolic abnormalities are present.^103^ However, after birth, these mice exhibit islet beta-cell failure and hyperglycaemia occurs within a few days followed by diabetic ketoacidosis and subsequent death. This demonstrates that insulin is not crucial for prenatal growth but is for postnatal metabolic homeostasis in mice. Insulin receptor knockout or suppression in individual organs in mice results in severely impaired glucose uptake in skeletal muscle; decreased fat storage in adipose tissue with normal levels of glucose and fatty acids in circulation; increased gluconeogenesis in the liver accompanied with systemic insulin resistance, hyperinsulinemia and glucose intolerance; decreased islet beta-cell proliferation and insulin secretion, and insulin receptor knockout in neurons increase appetite.^104^

Insulin-like growth factors (IGF-1 and IGF-2) are hormones that share homology with insulin and have been shown to activate the insulin receptor, and insulin can also bind to IGF receptors (IGF-1R and IGF-2R).^105^ Insulin receptor and IGFRs exhibit a high affinity for their ligand but can also bind each other’s ligands but with a much lower affinity.^40^ However, the physiological effect of insulin and IGF-1 binding to each other’s receptors has not been well studied. Both IGF-1R and insulin receptor activation promote anabolic processes in various tissues so it is reasonable to assume that when insulin binds to IGF-1R and IGF-1 binds to the insulin receptor, they induce the anabolic processes usually associated with these receptors binding their main ligands.^40^,^105^ However, given that IGF-1R and insulin receptor have decreased affinity for each other’s main ligand in comparison to their main ligand, it is likely that insulin and IGF-1 cannot stimulate the anabolic effects of each other’s receptor activation to the same degree as the main ligands for insulin receptor and IGF-1R at physiological concentrations.

#### Glucagon

**Figure 2:**
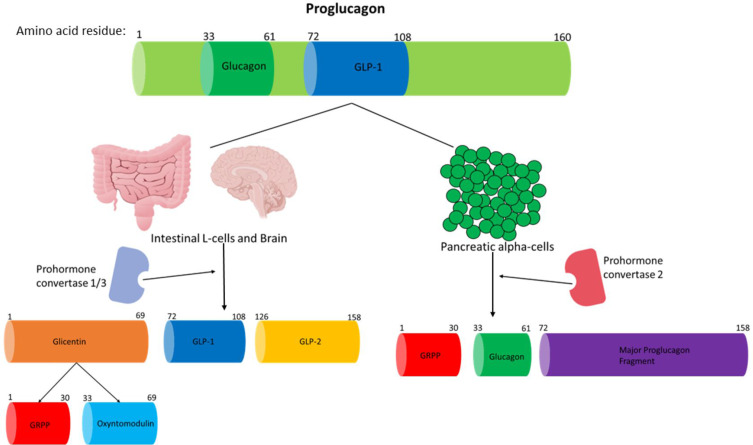
A summary of how proglucagon is alternatively processed in different tissues to produce the desired products. Proglucagon undergoes alternative processing by prohormone convertase enzymes in islet alpha-cells, L-cells of the gut and neuronal cells. Glucagon and GLP-1 are both pivotal hormones needed to maintain metabolic homeostasis. Only the biologically active products are shown and just GLP-1 and glucagon activity are discussed in this review. This figure and the information in its legend are adapted from these studies.^26^,^107^,^108^,^112^

Glucagon is a 29aa hormone that is produced by the preproglucagon gene.^11^ The preproglucagon gene is located on chromosome 2 (2q36-q37) spanning an estimated 9.4kb comprising of six exons and five introns, and exons 2 to 5 encode the proteins with known biological roles: glucagon is encoded in exon 3.^106^ The preproglucagon gene is transcribed to mRNA and then translated to form the preproglucagon protein (180aa residues), which is then cleaved in pancreatic alpha-cells, gut endocrine L-cells and neurons in the caudal brainstem and hypothalamus.^11^ Proglucagon (160aa residues) is then produced in these tissues by the removal of the signal peptide from the preproglucagon protein.^11^ In islet alpha-cells, prohormone convertase 2 modifies proglucagon to produce glucagon, which is formed by the removal of aa residues adjacent to 33 and 61 of proglucagon.^26^,^107^ Figure 2 summarises how proglucagon is processed in different tissues.

**Figure 3:**
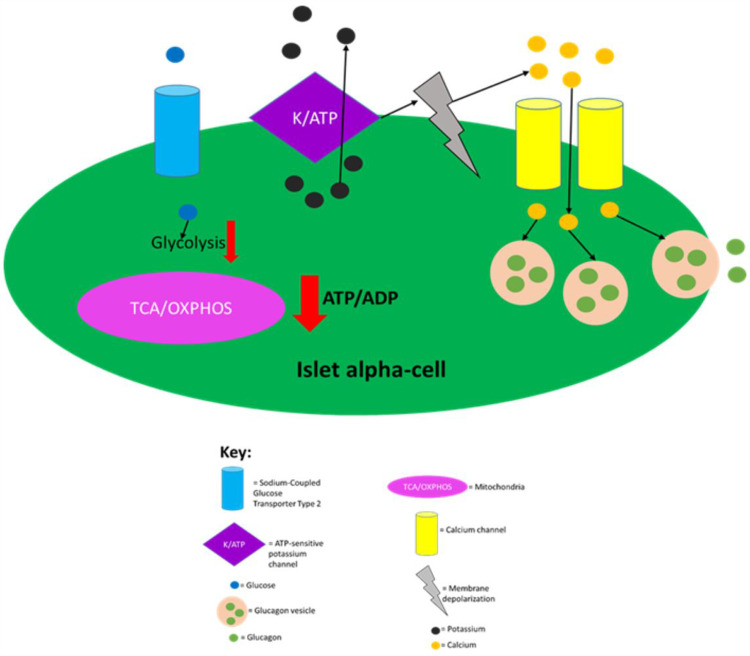
Regulation of glucagon secretion in pancreatic alpha-cells. Sodium-coupled GLUT2 receptor allows glucose to enter islet alpha-cells in an inuslin independent manner. When glucose is absent or at low levels in circulation, glycolysis and mitochondrial ATP production is greatly decreased leading to a decreased intracellular ATP/ADP ratio. This then allows for ATP-dependent K^+^ channels to be open, as ATP molecules block this channel. Opening of the ATP-dependent K^+^ channels then allows for intracellular potassium efflux which causes membrane depolarisation to an electrical range that permits opening of Ca2+ channels, which in turn, allows for extracellular calcium influx. The raised intracellular calcium levels then trigger glucagon secretion by causing glucagon vesicles to fuse with the plasma membrane. This figure and the information in its legend are adapted from these studies.^34^,^110^,^111^

Islet alpha-cells detect the absence of or low levels of glucose in circulation via hormone-insensitive sodium-coupled GLUT2, which results in the generation of action potentials of Na^+^ and Ca^2+^.^34^ The generation of action potentials results in the influx of calcium and exocytosis of glucagon granules.^110^ Similar to beta-cells, alpha-cells rely on ATP-dependent K^+^ (KATP) channels to play a key role in promoting exocytosis of intracellular granules, as they couple flux of glucose concentration in circulation to changes in membrane potential and calcium influx.^34^,^110^ At low glucose concentrations in circulation, alpha-cells have a low ATP/ADP ratio, which then permits ATP-dependent K^+^ channels to be open allowing for the efflux of potassium.^34^,^110^,^111^ Figure 3 summarises the regulation of glucagon secretion.

Additionally, the nervous system is also important for glucagon release. Hypoglycaemia results in the sympathetic and parasympathetic nervous systems mediating glucagon secretion from islet alpha-cells.^113^ The nervous system also inhibits glucagon secretion indirectly through the insulin secretion that it induces during the fed state.^60^ When blood glucose concentration rises, this then raises the intracellular ATP/ADP ratio in islet alpha-cells. The ATP then blocks the channel which depolarises alpha-cells to a membrane potential range where channels involved in action potentials are inactivated, meaning that calcium influx and subsequent exocytosis of glucagon vesicles no longer occur.^34^

Once released into circulation, glucagon mediates its effects on target tissues by binding to its receptor (glucagon receptor [GCGR]) on target tissues. GCGR is a class B G-protein-coupled receptor (GPCR) consisting of 485aa.^34^ GCGR is expressed in the liver, brain, kidney, GIT, pancreatic alpha and beta-cells, heart and adipose tissue.^34^,^114^ During the fasting state, glucagon promotes gluconeogenesis in hepatocytes by inducing glycogen breakdown.^114^ The glucose produced by this glycogen breakdown is exported into circulation which ensures sufficient plasma glucose levels so that glucose-dependent organs can produce enough ATP for their biological processes.^4^,^115^ It has been elucidated that glucagon acts as a positive autocrine regulator of islet alpha-cell function by inducing exocytosis of glucagon vesicles by acting on GCGR present on the surface of these cells.^116^ Exocytosis from islet alpha cells was blocked by a GCGR antagonist after glucagon administration.^116^ Studies have shown that glucagon action reduces fatty acid synthesis in adipose tissue and the liver, and additionally, this hormone induces lipolysis causing fatty acids to be released into the circulation from these tissues, which then enables them to be transported to the target tissues such as skeletal muscle to be catabolised to generate ATP when required.^117^ Interestingly, glucagon also exerts insulinotropic effects on islet beta-cells, and this is likely to allow for tissues to uptake glucose and fat once glucagon has induced their catabolism from nutrient storage (as tissues such as skeletal muscle rely on insulin for nutrient uptake from circulation), or the insulinotropic effects of glucagon could simply serve as a feedback inhibition mechanism.^114^,^118^ One study demonstrated the insulinotropic effect of glucagon, as when GCGR was blocked with an antagonist the insulin release was approximately halved in response to 10mmol glucose.^118^ This shows that insulin’s negative effect on glucagon secretion during the fed state likely prevents hyperinsulinemia.

It has been shown that glucagon can bind to GLP-1R, with a 100–1000-fold decreased affinity than GLP-1, promoting insulin release due to its homology with GLP-1. However, the insulinotropic effect of glucagon was confirmed not to be mediated by glucagon binding to GLP-1R here, as glucagon-enhanced insulin secretion was greatly dampened by the antagonism of GCGR.^119^ As glucagon can bind to GLP-1R, it may also be able to weakly stimulate insulin secretion through the action of this receptor as well as promote apoptotic resistance, proliferation and increased glucose sensing, as all of these effects are associated with GLP-1R activation.^107^,^120^ The strongest evidence that glucagon can induce insulin release via GLP-1R comes from rodent studies investigating the effect of GLP-1R antagonism and knockout.^121^ Glucagon’s effect on bone is not particularly well studied. However, studies from the 1970s have demonstrated that glucagon had anabolic effects on bone in patients with Paget’s disease.^122^ Recently, glucagon was also reported to have a positive effect on bone metabolism in T2D patients implying that this hormone increases the rate of skeletal modelling.^123^ Curiously, glucagon has been shown to induce satiety in both human and rat studies,^124^ which is surprising as this hormone is released into circulation due to a decrease in plasma levels of nutrients, and thereby it induces the breakdown of stored nutrients so that tissues throughout the body have fuel to generate ATP when needed.^114^ Therefore, it is reasonable to assume that glucagon would act to increase appetite, but, in reality, the opposite is the case.

Interestingly, studies have revealed that loss of function mutations in GCGR results in alpha-cell hyperplasia which results in glucagon cell adenomatosis in humans.^125^,^126^ Glucagon cell adenomatosis also occurs in patients without GCGR mutations, but these individuals usually have fewer and smaller tumours, as well as less islet alpha-cell hyperplasia.^125^ GCGR knockout mice produced in one study had islet alpha-cell hyperplasia and these mice were additionally shown to have large increases of glucagon and GLP-1 levels in circulation, but interestingly they had lower plasma glucose levels throughout the day, similar insulin levels and improved glucose tolerance compared to GCGR+ control animals.^127^ GCGR knockout is detrimental in mice as 50% did not survive until birth in this study. Furthermore, these mice exhibited delayed islet beta-cell differentiation and perturbed proportion of beta- to alpha-cells in embryonic islets. In adults, alpha-cell progression to maturity was hindered, the mRNA levels of several beta-cell genes (GLUT2, pancreatic duodenal homeobox-1 (PDX-1), and Maf-A) involved in insulin production and secretion were decreased, and there was an increase in the number and rate of proliferation of islet alpha-, beta- and delta-cells. Similar observations were found in GCGR knockout Zebrafish.^128^ These findings demonstrate that glucagon activity regulates the proportion of the different endocrine cell types in islets, the number of islets in the pancreas, and the development of the mature alpha-cell phenotype. GCGR knockout mice had unusually high levels of plasma GLP-1, which was shown to be due to increased L-cell number induced by GLP-1 action, even though L-cells do not express GLP-1R^129^. This suggests that GLP-1 action on L-cells is mediated through paracrine and/or neuronal signals. Additionally, glucagon has been shown to have anti-apoptotic effects on hepatocytes stimulated by Fas ligand activation and different experimental models of hepatotoxicity, and GCGR knockout results in increased hepatic susceptibility to apoptotic injury.^130^

**Figure 4:**
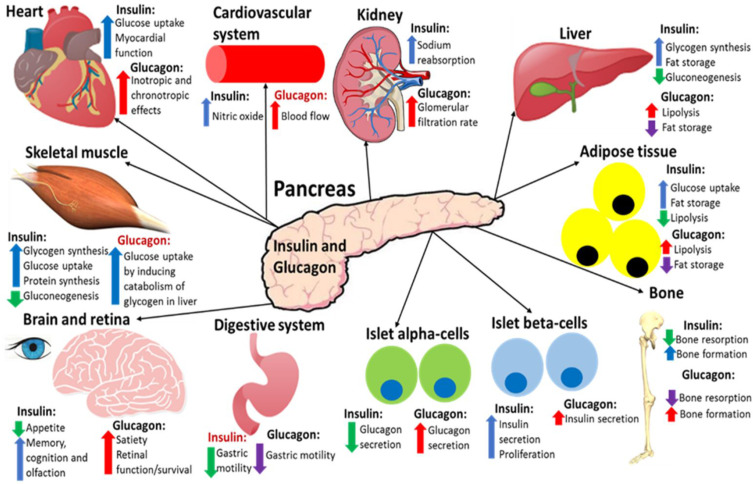
A summary of the effects that insulin and glucagon have on various organs after they have been released in response to the fed or fasting state, respectively. Both hormones have direct and indirect (highlighted red) effects on all of these organs. This figure and the information in its legend are adapted from these studies.^4^,^8^,^34^,^40^,^77^,^78^,^80^,^104^,^116–119^

In summary, insulin promotes the storage of ingested nutrients for the body to be able to utilise these nutrients when needed to generate ATP, and glucagon induces the breakdown of these stored nutrients when the body needs to utilise them during fasting or exercise.^4^ Figure 4 summarises the effects of insulin and glucagon on target tissues.

#### Somatostatin

Somatostatin, which is a hormone released by islet-delta cells, has been shown to have negative effects on both insulin and glucagon secretion.^131^ Additionally, somatostatin is also produced by endocrine cells in the central nervous system (resulting in decreased growth hormone secretion) and the GIT (resulting in slowed digestion).^131–133^ The half-life of somatostatin is <1 minute.^10^ Somatostatin receptors are known to be expressed in several tissues such as the GIT, brain, pancreatic islets, kidney, liver, spleen, heart, adrenal gland, immune system and lung.^131^,^134^,^135^ The release of somatostatin occurs similarly to insulin secretion from islet beta-cells, but somatostatin secretion is induced at plasma glucose concentrations as low as 3mM.^136^ Exogenously administered somatostatin inhibits glucagon release from islet alpha-cells and insulin release from islet beta-cells, and antibodies against somatostatin induce glucagon secretion from islet alpha-cells.^137^,^138^ Conflicting findings were reported when studies examined the local effect of somatostatin on glucagon release with isolated perfused pancreases.^139^,^140^

Exposing rat perfused pancreas to somatostatin receptor antagonists resulted in weakly enhanced glucagon secretion in response to glucose, but strongly enhanced secretion in response to arginine.^141^ Somatostatin knockout mice were initially reported to have a moderate phenotype, and most notably there were changes in the release of their pituitary hormones.^142^ However, a more recent study has shown that arginine-induced release of glucagon and insulin is significantly stimulated in mice due to the deletion of the somatostatin receptor gene, but basal insulin and glucagon levels were not affected.^143^ Interestingly, somatostatin knockout mice did not exhibit suppressed glucagon secretion from islet alpha-cells upon rising glucose concentrations in this study. Basal levels of insulin and glucagon were similar between somatostatin knockout mice and controls. However, after stimulation with 20mM glucose, somatostatin knockout mice had increased plasma insulin and glucagon levels. The inhibition of GSIS by lowering glucose levels was similar though between somatostatin knockout and wild-type mice. One study reported that somatostatin does not suppress insulin secretion by acting on the central nervous system but acts peripherally on islet beta-cells.^144^ It is correct to state that somatostatin does reduce the incretin effect as it suppresses GIP and GLP-1 release from the GIT, giving it an indirect role in dampening insulin secretion.^145^ These findings suggest that physiological concentrations of somatostatin reduce glucagon secretion during low glucose and insulin secretion during high glucose levels. A recent study demonstrated that in mice somatostatin and glucagon secretion are linked in a reciprocal feedback cycle with somatostatin inhibiting glucagon secretion at low and high glucose levels, and glucagon stimulating somatostatin secretion via the glucagon and GLP-1 receptors.^146^ One study demonstrated that somatostatin also likely mediates satiety.^147^ The effect of the nervous system on somatostatin release has not been well studied in humans but it has been shown that the vagal nerve has inhibitory effects on isolated perfused rat and pig pancreases.^148^

#### Pancreatic Polypeptide

Pancreatic polypeptide is produced by islet gamma-cells during the postprandial period, peaking at 15–30 minutes which is followed by a lower sustained phase lasting for up to 6 hours after nutrient ingestion.^149^ Receptors for pancreatic polypeptide have been detected in the hypothalamic arcuate nucleus located in the brainstem, and more recently pancreatic polypeptide has also been shown to act on a different receptor expressed in islet alpha-cells.^150^,^151^ The vagal nerve is the main stimulator of pancreatic polypeptide secretion during the postprandial period.^152^ In healthy humans, pancreatic polypeptide has been shown to weakly increase basal insulin secretion by unknown mechanisms but it does not seem to have any effect on GSIS.^153^ In contrast, another study found that pancreatic polypeptide inhibited GSIS in rat and human beta-cell lines and isolated mouse islets.^154^ In this study native pancreatic polypeptide also protected against beta-cell apoptosis. Direct exposure of pancreatic polypeptide to mouse islets suppressed glucagon release from islet alpha-cells.^150^ Another study found that pancreatic polypeptide decreases somatostatin secretion in mice and human islets.^155^ Pancreatic polypeptide deficient patients, as a result of pancreatic resection or chronic pancreatitis, exhibit reversed hepatic insulin resistance upon pancreatic polypeptide infusion.^156^,^157^ Comparable observations were found in animal models of chronic pancreatitis or pancreas resection.^158^ Pancreatic polypeptide is thought to be important in inducing satiety as pancreatic polypeptide plasma levels are nearly eliminated in obese children with Prader-Willi syndrome. Bovine pancreatic polypeptide infusion has reduced food intake in both Prader-Willi syndrome patients and healthy humans.^159–161^ Pancreatic polypeptide was also shown to reduce food intake in *ob/ob* mice and increase energy expenditure.^162^ Pancreatic polypeptide is known to reduce pancreatic secretion to slow digestion but the effects on the rest of the GIT in humans are not currently clear.^163^,^164^

#### Ghrelin

Ghrelin is produced by the stomach and the islet epsilon-cells during the pre-prandial period and it has a half-life of ~30 minutes.^165^,^166^ The nervous system also modulates ghrelin secretion, as sympathetic nerve excitation stimulates ghrelin secretion from the GIT.^167^ The effect of the nervous system on ghrelin release from the pancreas is not well-studied.^166^ The main action of ghrelin is to induce hunger by acting on hypothalamic brain cells expressing the ghrelin receptor.^168^ It has been elucidated that by acting on these cells, ghrelin increases gastric acid secretion and gastrointestinal motility also to prepare for nutrient ingestion.^169^ Ghrelin also mediates insulinostatic actions even though human beta-cells do not express any known receptors for ghrelin, but a recent study discovered that the ghrelin receptor is expressed solely by delta-cells, implying that ghrelin dampens insulin secretion by inducing somatostatin release.^170^ However, in rodents, ghrelin mediates insulinostatic actions via direct mechanisms.^171^

Treatment with ghrelin does not affect insulin secretion at 5.5mM glucose but significantly reduces it (by ~30%) at 16.8mM glucose. Given that ghrelin should not be produced during the fed state, it is unlikely that it modulates insulin secretion associated with the postprandial period. However, it may dampen any minimal insulin secretion during fasting given its insulinostatic actions, which would indirectly assist glucagon’s ability to release nutrients from storage. Interestingly, it has been elucidated that ghrelin’s insulinostatic actions are accompanied by a simultaneous increase in plasma glucose levels and the enhanced ability of insulin to suppress glucose production in ghrelin knockout mice has been reported, but it is not clear how this was achieved mechanistically.^172^ Hence, it seems likely that ghrelin induces glycogenolysis and/or inhibits glucose uptake into peripheral tissues from circulation via direct mechanisms. A more recent study found that ghrelin inhibited GSIS in islets from non-T2D and T2D donors and that ghrelin mRNA expression and fasting circulating ghrelin levels were lower in T2D patients.^173^ The findings from this study further support ghrelin’s ability to mediate insulinostatic actions, and that reduction in plasma ghrelin could either promote T2D pathogenesis or be an adaptive response to stop further reduction in insulin secretion in T2D patients. Results from studies indicate that ghrelin also promotes lipid retention in adipocytes, which seems paradoxical given this hormone’s role as a hunger hormone, as during starvation lipids need to be released from stores and catabolised for ATP production, although it has also been argued that ghrelin protects fat stores from being utilised so that they can be used for ATP production during prolonged starvation.^174^,^175^

### Incretin Hormones

In the latter half of the nineteenth century, understanding the external and internal secretion mechanisms of the pancreas became a focus for European physiologists. It was noticed in this period that the pancreas was the origin of diabetes mellitus.^176^ In 1906, after the discovery of secretin, it was first tested if the gut aided the pancreas with disposing of nutrients via stimulation of pancreatic internal secretion by Moore and colleagues, who orally administered porcine small intestinal extract to treat diabetic patients.^177^ The results of this oral intake were not only negative but also inconclusive, which would be expected due to the lack of the modern-day understanding that proteins are proteolytically degraded in the stomach. After the discovery of insulin in 1922, new attempts were made to investigate the influence of the intestinal mucosa on blood glucose concentration.^176^ In 1928, it was demonstrated by Zunc and LaBarre that the injection of secretin, which is extracted from the small intestinal mucosa, shows a hypoglycaemic effect that is mediated by the pancreas.^178^ The name incretin was then developed by LaBarre to describe a substance that only causes hypoglycemia but does not promote pancreatic exocrine secretion.^179^ For the next few decades, false-negative conclusions were drawn from several subsequent studies, which stated that the existence of an incretin hormone was unlikely, hindering any further investigation into this area.^176^ During the 1960s, it was demonstrated that orally administered glucose promotes markedly greater pancreatic insulin secretion than that induced by intravenously administered glucose, despite the plasma glucose levels being very similar- this was termed the “incretin effect”.^11^,^180–182^

GIP is secreted by K cells in the small intestine. It was first isolated, as an inhibitor of gastric acid secretion, from porcine small intestine crude extracts.^13^,^183^ Subsequently, it was discovered that GIP could also promote insulin secretion in animals and humans, and henceforth, it was named glucose-dependent insulinotropic polypeptide.^11^,^184^ In 1981, antibodies against GIP were demonstrated to not stop the incretin effect.^185^ Another incretin hormone was then discovered, GLP-1, which was first identified in the translational products of mRNAs isolated from anglerfish pancreatic islets.^186^ Subsequently, it was shown that hamster and human preproglucagon cDNAs encode GLP-1 and −2, but only GLP-1 displayed incretin activity.^187^ The incretin hormones play a crucial role in maintaining metabolic homeostasis via augmentation of insulin secretion from islet-beta-cells: the incretin effect accounts for 60–70% of total insulin released from the pancreas during the postprandial period.^17^ It is estimated that GIP and GLP-1 mediate 60 and 40% of the incretin effect, respectively.^16^ However, islet beta-cells can produce and secrete insulin in an incretin-independent manner as long as blood glucose levels are 5mM or higher.^4^,^17^ Both GIP and GLP-1 mediate their actions by binding to their specific GPCRs on target tissues: GIP binds to the GIP receptor (GIPR) and GLP-1 binds to GLP-1R.^120^ It has been demonstrated that genetic ablation of GIPR and GLP-1R either individually or at the same time in mice results in impaired insulin secretion confirming that both GIP and GLP-1 mediate the incretin effect.^188^

#### GLP-1 Production and Secretion

GLP-1, which is a 30aa-long incretin hormone, is secreted by enteroendocrine L-cells present in the distal ileum and colon of the GIT in response to postprandial food intake.^107^ GLP-1 is formed by alternative processing of the preproglucagon by prohormone convertase 1/3 (Figure 3).^11^ Exon 4 encodes GLP-1.^106^ GLP-1 is also generated similarly in the central nervous system.^107^,^108^ Although the pancreatic beta-cells are the well-characterised target for GLP-1, the incretin hormone also targets multiple other organs.^107^ GLP-1’s ability to mediate the incretin effect is its most studied and significant physiological effect due to the use of GLP-1 analogues (exenatide and liraglutide, lixisenatide, dulaglutide, albiglutide and semaglutide) in T2D treatment.^26^,^107^ The targeting of GLP-1 physiologically on organs other than the pancreas has not been well studied but it is becoming increasingly clear that this hormone does modulate the biological activity of a variety of organs throughout the body. GLP-1 mediates its actions by binding to its receptor (GLP-1R), which is localised at the cell surface, in target tissues.^11^,^26^

After GLP-1 has been produced from proglucagon, its stability is improved by the conversion of the carboxyl group of 36th aa (arginine) to an amide (GLP-1 [1–36 amide]).^107^ Before secretion, 1–6aa are cleaved from GLP-1 (1–36 amide) and GLP-1 (1–37) to form GLP-1 (7–36 amide) and C-terminal amidated GLP-1 (7–37). It is estimated that GLP-1 released into the bloodstream contains 80% in the GLP-1 (7–36 amide) form and the remaining 20% is in the GLP-1 (7–37) form.^26^ The two versions of GLP-1 are ~50% homologous with glucagon,^189^ and they both have similar affinity for the GLP-1R and also exhibit similar potency.^26^ Nevertheless, it has been suggested that GLP-1 (7–36 amide) has greater stability in the circulation so there may be a physiological reason as to why GLP-1 is produced in two forms.^11^

**Figure 5:**
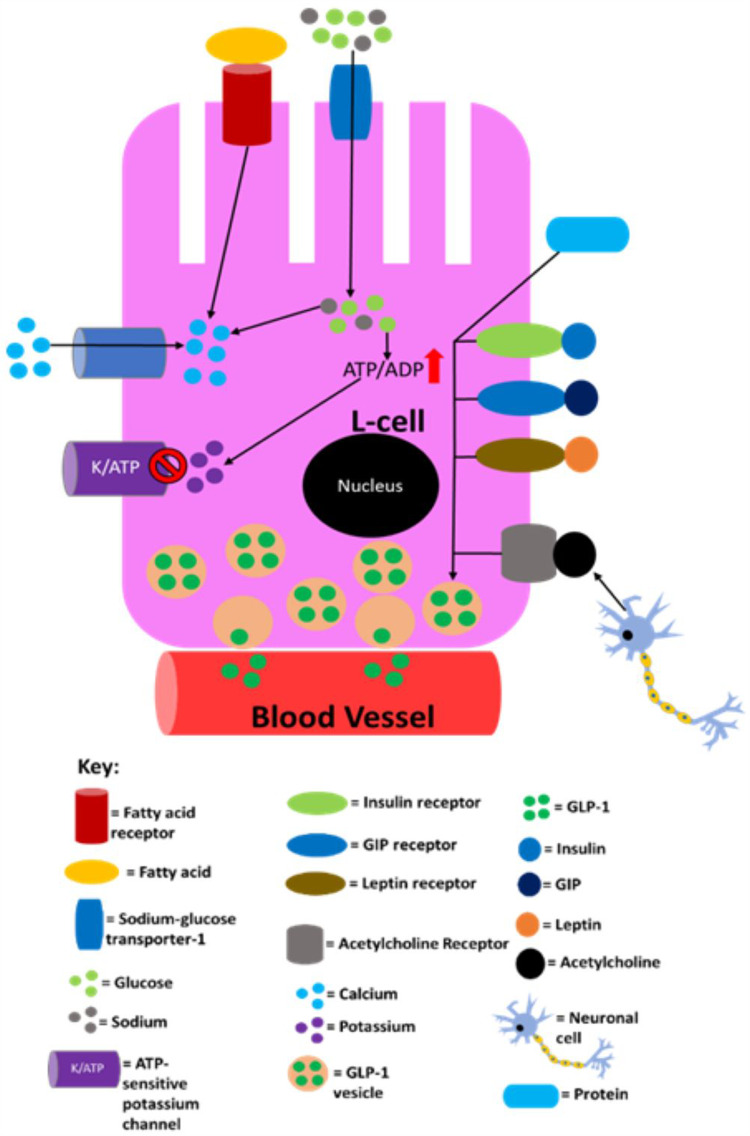
A summary of the processes in L-cells that are known to lead to/may lead to GLP-1 secretion into the circulation. Sodium entry into the L-cell promotes calcium influx by inducing depolarisation and glucose and fatty acids also induce calcium influx by raising ATP levels as a result of their catabolism. The now elevated calcium levels promote exocytosis of GLP-1-containing vesicles, resulting in GLP-1 being released into circulation. Proteins also promote GLP-1 release, but it is not currently mechanistically understood how. This figure is reproduced from (Reed J, Bain S, Kanamarlapudi V. Recent advances in understanding the role of glucagon-like peptide 1. *F1000Research*. 2020; 9(239):1–14; Creative Commons)^32^ and the information in its legend are adapted from these studies.^195–200^

It has been established that there is a positive correlation between GLP-1 levels in the circulation and the amount of nutrients L-cells are exposed to.^190^ Initially, GLP-1 secretion by L-cells was believed to occur in a glucose-dependent manner. However, it has been shown that GLP-1 secretion in response to mixed nutrient (carbohydrates, fats and proteins) intake is much higher than that secreted after glucose ingestion.^191^ This suggests that fats, proteins and glucose have a synergistic effect on GLP-1 secretion. Additionally, it has been shown that fats and proteins can promote GLP-1 secretion in the absence of glucose.^190^ Ingestion of nutrients has also been shown to increase GLP-1 expression at mRNA level in L-cells.^192^ In the postprandial period, GLP-1 levels rise in the circulation to 40–60pM from 5–15pM during fasting.^26^,^193^ After food ingestion, the GLP-1 response is initiated within 15 minutes and reaches the maximum in 30 minutes.^55^,^190^,^194^ It is currently elusive why the GLP-1 response occurs so rapidly but it is speculated that L-cells in the upper jejunum and the vagal nerve play a role.^107^ However, the response occurs after the “cephalic phase” of insulin secretion implying that any neuronal signals that promote insulin release do not induce GLP-1 secretion.^26^,^107^ Although neuronal regulation of GLP-1 secretion is not well-studied in humans, studies using rodents have found that GLP-1 secretion from L-cells in response to GIP stimulation is induced by the nervous system. This seems plausible given that L-cells express receptors that participate in neuronal signalling.^145^,^195^ L-cells have been shown to express receptors for metabolic hormones including, but not limited to, insulin, leptin and GIP, although the degree to which leptin and insulin stimulate overall GLP-1 secretion is currently elusive/unclear. GIP-mediated GLP-1 release has been shown to occur in rodents via acetylcholine release by the enteric nervous system.^195–200^ Supraphysiological concentrations of GIP could potentially activate GIPRs on L-cells, enhancing GLP-1 secretion. Figure 5 summarises the regulation of GLP-1 secretion.

The half-life of GLP-1 in the circulation is between 1–2 minutes.^107^ Dipeptidyl peptidase-IV (DPP-IV) is the enzyme responsible for the majority of the rapid GLP-1 degradation, by cleaving the first two N-terminal residues from GLP-1 (7–36 amide) and GLP-1 (7–37) to form GLP-1 (9–36 amide) and GLP-1 (9–37), respectively.^11^,^107^ After both forms of GLP-1 have been exposed to DPP-IV, they are either rendered inactive or become an antagonist for the GLP-1R.^107^ Interestingly, only an estimated 15% of the active GLP-1 reaches the portal vein before the liver, due to the rapid inactivation through DPP-IV.^26^,^107^ Therefore, it is thought that ~85% of GLP-1 present in the circulation is in an inactive form.^26^ From a bioenergetics angle, the GLP-1 response is highly inefficient (GLP-1 synthesis requires ATP), due to the majority of the GLP-1 secreted being inactivated before it reaches its targets.^4^,^26^ However, recent findings suggest that the inactive forms of GLP-1 may have actions similar to that of insulin on the liver, vasculature and heart given the findings from rodent and in vitro studies.^201^,^202^ Therefore, the “inactive forms” may not be inactive and act on signalling pathways that are currently unidentified. This is a plausible explanation since evolution is very unlikely to develop such an ATP wasteful response. It has even been indicated that all GLP-1 released by L-cells is inactivated before it reaches the pancreas and that GLP-1 mediated insulin release is induced by local paracrine actions in the islets during the postprandial period, and there is emerging evidence that this is the case, which would mean that GLP-1 released by L-cells only acts on its receptor (GLP-1R) expressed on nerve terminals of neighboring vagal afferents, conveying signalling to the solitary nucleus of the hindbrain to promote satiety, delay gastric emptying and suppress hepatic glucose production.^107^,^203^ The physiological relevance of GLP-1 metabolites remains an area of controversy.^204^

#### GLP-1 Targets and Effects

GLP-1 mediates its effects via its receptor GLP-1R, which is a GPCR consisting of 463aa.^26^ GLP-1R expression occurs in not only the pancreas alpha and beta-cells but also in the GIT, cardiovascular system, kidney, lung and the central and peripheral nervous system.^11^,^107^ The signalling pathways coupled to GLP-1R activation vary in different tissues, which generates the desired physiological effects in each tissue.^107^ GLP-1 also affects organs indirectly by potentiating insulin secretion. It accounts for ~28% of the total postprandial insulin released into circulation.^17^,^108^

##### Pancreatic Islet Alpha- and Beta-Cells

Since GLP-1 analogues play a vital role in the treatment of T2D, the ability of this hormone to mediate the incretin effect is its most studied and most clinically relevant action.^32^,^109^,^112^ GLP-1 analogues effectively reduce hyperglycaemia in patients as they have a much longer half-life than native GLP-1, prolonging the incretin effect in patients.^32^,^112^ The ability of GLP-1 to stimulate insulin secretion is dependent on high glucose levels, as this hormone does not induce insulin secretion in the presence of low blood glucose.^11^ Additionally, GLP-1R activation also induces the translocation to the nucleus of the PDX-1 transcription factor, giving GLP-1 a role in islet beta-cell survival.^112^,^205^,^206^ Interestingly, chronic liraglutide administration to diabetic mice that had been injected with alloxan prevented the loss of beta-cell mass, which was shown to be due to both an increase in beta-cell proliferation and a decrease in apoptosis.^207^ Liraglutide treatment was also demonstrated to inhibit beta-cell apoptosis in isolated human pancreatic beta-cells, and after 24 hours, the islet beta-cell proliferation rate tripled.^208^ In streptozocin-induced type 1 diabetic rats and isolated human pancreatic ducts, exendin-4 administration was also demonstrated to increase the population of islet beta-cells.^209^ Endogenous GLP-1 has also been suggested to promote beta-cell proliferation in rodent cell lines and isolated rodent islet cells in addition to inducing apoptotic resistance.^206^,^210^ Additionally, activation of GLP-1R has been demonstrated to promote cell survival during glucotoxic and lipotoxic conditions, excessive nitric oxide levels, Ca^2+^ depletion, oxidative stress, and cytokine-induced endoplasmic reticulum stress in primary beta-cells and cell lines.^211–215^

Recently, GLP-1 action has additionally been expanded to potential regulation of autophagy in islet beta-cells: Exendin-4 induced autophagy in INS-1E cells and isolated human islets during chronic exposure to excess nutrients, by promotion of autophagosomal-lysosomal fusion.^216^,^217^ Exendin-4 was also demonstrated to increase lysosomal function in another study, whereby autophagosome clearance in a rat model of tacrolimus-induced diabetes was increased, promoting cell survival given that autophagosome accumulation causes intracellular damage.^218^ Additionally, in this study, in vivo exendin-4 treatment also decreased tacrolimus-induced hyperglycemia, oxidative stress, and apoptosis further demonstrating mechanisms by which exendin-4 promotes cell survival. Interestingly, recent studies have found that chronic GLP-1R activation results in enhanced ATP production and upregulation of glycolytic enzymes which increases the potential of ATP production anaerobically.^219^ It has been postulated that the ability of GLP-1 to enhance metabolism may decrease endoplasmic reticulum stress via increasing mitochondrial production of ATP and Ca^2+^, which could be utilised for assisting endoplasmic reticulum homeostasis.^206^ Interestingly, chronic GLP-1R activation activates distinct signalling pathways; GLP-1R agonist treatment induced insulin-like growth factor-2 secretion and promoted expression of its receptor, which has been suggested to enhance the pro-survival actions of GLP-1 in islet beta-cells.^206^,^220^,^221^ Given that endoplasmic reticulum stress, impaired autophagy and proliferation, and increased apoptosis are all suggested to promote islet beta-cell pathology in T2D, and the findings that GLP-1R activation can influence all of these give GLP-1 and its analogues a role beyond just enhancing insulin secretion.^206^

GLP-1 has also been shown to reduce blood glucose levels via direct inhibition of glucagon secretion from islet alpha-cells in an insulin-independent manner. However, the underlying mechanism for GLP-1 accomplishing this is not known, as whether or not GLP-1R is localised to the islet alpha-cell surface is a matter of debate.^108^ The strongest evidence that demonstrates GLP-1’s ability to directly inhibit glucagon secretion comes from the observation that the ability of T1D patients to secrete glucagon is suppressed after GLP-1 infusion and glycaemic control is improved.^222^ GLP-1 is however known to indirectly inhibit glucagon release through the insulin section it induces.^223^ Recently, a study found that alpha-TC1/6 cells can secrete GLP-1 under hyperglycemic, hyperlipidemic or inflammatory conditions during in vitro experiments, suggesting that this hormone may act locally to influence both islet alpha- and beta-cell behaviour during pathological conditions.^224^ Although, there is evidence to suggest that islet alpha-cells can secrete GLP-1 during healthy circumstances and that this secretion becomes elevated during diabetic/disease conditions, whether these cells secrete GLP-1 and the physiological relevance of this remains unclear.^225^ Interestingly, another study found that liraglutide treatment was shown to increase alpha-cell GLP-1 expression in a beta-cell GLP-1R-dependent manner in human islets.^226^ This implies that GLP-1 can induce alpha-cells to produce GLP-1 via indirect mechanisms. Despite the debated GLP-1R expression in islet alpha-cells, recent studies have provided evidence that GLP-1R agonists may mediate direct effects on alpha-cells via GLP-1R action.^11^,^227^,^228^ Islet alpha-cell GLP-1R knockout mice were unable to inhibit glucagon secretion at high glucose levels, and surprisingly, these mice exhibited reduced glucagon secretion during low glucose conditions.^229^ A recent study also found that that glucagon’s ability to induce insulin secretion was reduced in isolated islets from GLP-1R knockout mice, and in wild-type mice a GLP-1R antagonist significantly reduced glucagon-induced insulin secretion.^122^ These data suggest that GLP-1 receptors contribute to the insulinotropic action of glucagon via GLP-1R signalling. An additional recent study found that GLP-1 action on human pancreatic islet alpha-cells was mediated independently of any paracrine signalling, as preventing insulin and somatostatin signalling did not affect the inhibition of glucagon secretion.^227^ Determining whether GLP-1 has direct/indirect effects on islet alpha-cells and what receptor(s) mediate these actions requires further investigation.

##### Gastrointestinal Tract (GIT) and Food Intake

In the GIT, GLP-1 reduces gastric motility, inhibits postprandial gastric acid secretion, decreases postprandial pancreatic exocrine secretion and reduces smooth muscle activity in the small intestine,^194^ promoting a slower digestion of nutrients from the GIT.^107^ These effects all result in a slower and steadier uptake of nutrients into the circulation; preventing hyperglycaemia and the requirement of a fast and aggressive insulin response.^11^,^107^ Thus, GLP-1 also plays an important role in the digestion process (this may be the major action of short-acting GLP-1RAs such as lixisenatide) as well as potentiating the incretin effect. It has been demonstrated that the inhibitory action of GLP-1 on the GIT is mediated through a vagal pathway.^229^ Subsequently, evidence of the ileal-brake activity of GLP-1 was further provided using the GLP-1R antagonist exendin_9-39_.^230^ GLP-1 has also been shown to induce satiety, although how it does so mechanistically has not yet been determined.^107^,^231^ It was initially postulated that GLP-1’s effect on appetite may be induced by its ability to reduce gut motility; however, it has been shown that GLP-1 has direct effects on certain neurons in the hypothalamus.^194^ GLP-1 is expressed in neurons of the brainstem and GLP-1R is present in the hypothalamic regions that regulate energy homeostasis and appetite.^11^,^107^ Intracerebroventricular injection of GLP-1 induces satiety in rats, and this is reversed by exendin_9–39_ demonstrating that GLP-1 mediates its actions via its receptor here.^232^,^233^

Studies found that intracerebroventricular administration of GLP-1 in rodents can mediate satiety with no food present in the GIT and when gastric emptying is prevented; therefore, in rodents, GLP-1 induces satiety by its actions on caudal brainstem neurons.^11^,^107^,^194^ The mechanisms which enable peripheral GLP-1 to induce satiety are not understood, but it is thought that GLP-1 binding to GLP-1R on neurons in the GIT, hepatoportal bed and CNS regulate this effect.^107^,^234^ The varying weight loss observed for the different GLP-1 analogues currently used for T2D treatment has been suggested to be due to their ability to penetrate the CNS.^197^,^200^ It is plausible that GLP-1 directly decreases appetite, as this would allow ingested nutrients to be digested and prevent hyperglycaemia/hyperlipidaemia or undesirable nutrient storage by more ingestion of nutrients. It is now widely accepted that liraglutide therapy effectively promotes weight loss in obese individuals with/without T2D by reducing food intake via direct action on neurons in the brain.^235^ In recent years, both liraglutide and semaglutide have been approved in the USA to treat obesity given their ability to induce weight loss to a greater extent than other drug options and exhibit superior safety, and in both diabetic and non-diabetic individuals liraglutide and semaglutide have been recommended as the first-line drugs for obesity treatment.^236^

##### Cardiovascular System

During the 1990s, several cell types in the human cardiovascular system were found to express GLP-1R, such as vascular smooth muscle, cardiomyocytes, endocardium, and coronary endothelium/smooth muscle.^11^ Intravenous administration of GLP-1 has been reported to mediate multiple cardiovascular beneficial effects in animal models: improved left ventricular contractility, increased functional recovery and cardiomyocyte viability, reduced myocardial infarction, reduced atherosclerotic lesions, improved endothelial function, improved blood flow and decreased hypertension.^11^,^107^ In diabetic and non-diabetic humans with cardiovascular pathology such as coronary heart disease, recombinant native GLP-1 treatment has induced several cardiovascular benefits including reduced arrhythmias, improved left ventricular function, and improved endothelial function.^237^,^238^ Several studies using animal models have demonstrated that acute GLP-1 infusion increased heart rate, blood pressure and glucose uptake by the heart, whereas chronic GLP-1R agonism in rodents reduced blood pressure and prevented hypertension.^11^,^107^,^239^ These findings imply that GLP-1 mediates direct effects on the heart. Short-term human clinical trials demonstrated that acute GLP-1 therapy either increased heart rate and blood pressure or did not affect these.^11^,^239^

GLP-1 analogue and DPP-IV inhibition therapies decreased circulatory lipid levels in healthy and diabetic rodents, and similar therapies also produced the same effects on T2D patients.^197^,^239^,^240^ However, one study demonstrated that 24 weeks of exenatide administration did not influence lipid levels.^241^ Rodent and human studies have produced evidence that GLP-1 therapies have antiatherosclerotic and angiogenic effects, and these therapies hindered the development of diabetic cardiomyopathy.^11^,^242^,^243^ Long-term human studies demonstrated that chronic DPP-IV inhibition did not significantly mediate any cardiovascular (CV) benefits.^244^,^245^ However, chronic GLP-1-based therapies demonstrated several CV benefits in diabetic patients, as liraglutide and exenatide therapies decreased all-cause mortality.^239^ Liraglutide mediated a significant decrease in CV mortality whereas exenatide reduced nonfatal stroke in diabetic patients. A recent systematic review and meta-analysis of seven clinical trials found that GLP-1R agonist treatment reduces major adverse cardiovascular events by 12% overall in type 2 diabetic patients.^246^ The recent findings of the SELECT study also indicate the therapeutic potential of GLP-1 receptor analogues to improve cardiovascular outcomes in overweight and obese individuals without T2D, as weekly administered semaglutide was found to be superior to placebo in reducing the incidence of death from cardiovascular disease, nonfatal myocardial infarction and nonfatal stroke.^247^ Mechanistically, it is unclear how GLP-1-based therapies exert beneficial effects on the cardiovascular system, but studies have suggested that this is achieved by direct (cardiovascular GLP-1R activation) and indirect (promotion of the incretin effect) mechanisms.^11^,^239^

It is also possible that GLP-1 therapies mediate beneficial actions via extra cardiovascular GLP-1 signalling, as one study reported that exendin-4 administration in rat femoral arteries did not affect short-term triglyceride exposure-induced endothelial dysfunction.^248^ Interestingly, in 2008 a study found that the GLP-1 metabolite GLP-1 (9–36) afforded significant protection against ischemia-reperfusion injury in mice and induced vasodilation in a GLP-1R independent manner.^249^ Further, a study conducted in 2010 revealed that treatment of mouse cardiomyocytes with GLP-1 (9–36 amide) resulted in extracellular signal-regulated kinase phosphorylation, Akt activation and decreased apoptosis caused by hydrogen peroxide stress or hypoxia.^250^ This study highlights that after GLP-1 has been processed by DPP-IV it may still have physiological functions and is not inactivated as current dogma suggests in humans.

##### Immune System

Findings from multiple studies have demonstrated that GLP-1 also regulates the immune system.^11^,^112^ In rodents, GLP-1R mRNA is present in several immune cell types: macrophages, Treg cells, thymocytes, splenocytes, bone marrow-derived cells, and natural killer cells etc.^11^ Administration of GLP-1R agonists to humans or animals with T2D or obesity is usually associated with a reduction in local or systemic inflammation.^251^ When the GLP-1 analogue liraglutide was administered to patients with psoriasis (an inflammatory disease associated with obesity and diabetes) this led to a decreased psoriasis area severity index, and cytokine secretion was decreased from natural killer cells in a glycaemic control-independent manner.^252^ However, a subsequent study was not able to confirm that liraglutide therapy improved psoriasis prognosis.^253^ GLP-1 secretion from L-cells increases substantially in response to many cytokines such as interleukin-6, and interestingly, GLP-1 levels are elevated in patients with sepsis and are correlated with the severity of illness and clinical outcomes, although it is currently unclear if the elevation of GLP-1 levels exacerbates or alleviates pathology during sepsis.^254^,^255^

High-fat diet-fed mice treated with exendin-4 (a hormone found in the saliva of the Gila monster which is synthetically modified to produce exenatide for T2D treatment) had decreased mRNA levels of the pro-inflammatory cytokines tumour necrosis factor-alpha, monocyte chemoattractant protein 1 and signal transducer and activator of transcription 3.^256^ One study highlighted that GLP-1 plays an important role in the immune system: intraperitoneal injections of GLP-1 and gastrin were shown to restore normoglycemia in diabetic NOD mice (a type 1 diabetic mouse model) by increasing the pancreatic beta-cell mass and insulin content, reducing beta-cell apoptosis, and downregulating the autoimmune response.^257^ In GLP-1R KO mice, the distal gut was found to display microbial dysbiosis and was more sensitive to inflammation-related injury, which was thought to be due to dysregulated inflammation-related gene expression. This was substantially corrected following the transplantation of bone marrow from GLP-1R-positive mice into these GLP-1R knockout mice.^258^ Multiple studies have demonstrated anti-inflammatory actions, such as reduction of secretion of pro-inflammatory cytokines or increase in the secretion of anti-inflammatory cytokines, of GLP-1R agonists,^259^ which may have future therapeutic applications for both T1D and T2D, as well as inflammatory/autoimmune diseases.

##### Kidney

In 2004, it was found that GLP-1 mediates effects on the kidney: Healthy and obese individuals treated with GLP-1 exhibited increased sodium excretion, glomerular filtration rate and urinary secretion.^260^ GLP-1 downregulated Na+/H+ exchanger isoform 3 in the renal proximal tubule of rats, suggesting that GLP-1 could be used to treat hypertension and sodium retention disorders.^261^ Exendin-4 treatment also enhanced renal function, and reduced inflammation, fibrosis and proteinuria in rat kidneys, and these effects occurred in a glucose-lowering independent manner.^262^ A recent study found that liraglutide therapy resulted in significantly lower rates of renal outcomes among patients with type 2 diabetes who were at high cardiovascular risk.^263^ Multiple clinical studies over the last few years have found that several GLP-1R agonists have beneficial effects on diabetic kidney disease independent of their glucose-lowering actions mediated by natriuresis, anti-inflammatory and anti-oxidative stress properties, and suppression of renal fibrosis.^264^ Moreover, several studies have reported that GLP-1R agonists improve renal outcomes, especially in T2D patients who are at high risk of cardiovascular disease.^246^,^265^ The findings from all these studies suggest that GLP-1 improves kidney function and mediates protective effects on the kidney, meaning that it is a promising therapeutic option for diabetic kidney disease. However, further research is required to establish how to use these drugs to produce optimal outcomes for kidney pathology.^264^ The outcomes of the currently ongoing FLOW study will further inform the therapeutic potential of GLP-1R analogues to improve renal and cardiovascular prognosis in patients with renal impairment and T2D.^266^,^267^

##### Nervous System

GLP-1 has also been reported to affect the nervous system.^11^ Lateral ventricular administration of GLP-1 or exendin-4 in mice reduced endogenous levels of amyloid beta-protein (a protein that promotes Alzheimer’s disease), and infusion of GLP-1 and exendin-4 into rat hippocampal neurons also prevented cell death induced by amyloid-beta protein.^268^ GLP-1 administration to intracerebroventricular tissue improved hippocampal synaptic plasticity and reversed impairment in long-term potentiation induced by amyloid-beta protein, which was subsequently administered.^269^ One human study found that liraglutide therapy was found to increase cerebral glucose metabolism in patients with Alzheimer’s disease.^32^ In Parkinson’s disease, nigrostriatal neurons undergo cell death and GLP-1R is expressed in these neurons.^11^ Therefore, any undesirable GLP-1R activity could promote the pathology associated with Parkinson’s disease, or future therapeutic options may be able to regulate GLP-1R actions to delay or prevent the manifestation of this disease. Recent clinical studies have found that exenatide therapy was found to induce long-lasting improvements in motor and cognitive function in patients with Parkinson’s disease.^270^,^271^ GLP-1 or exendin-4 treatment of cultured rat embryonic cerebral cortical cells promoted cell survival during hypoxic injury, and this effect was blocked by GLP-1R antagonists and not seen in GLP-1R knockout mice.^272^ Exendin-4 also promoted apoptotic resistance and improved viability in NSC19 neuronal cells (a mouse cell line) during oxidative stress.^273^ Liraglutide was also shown to promote SH-SY5Y cell survival by shifting cell fate from apoptosis to survival under chronic stress conditions.^274^

Additionally, a recent study found that GLP-1 treatment improves learning and memory in type 2 diabetic rats.^275^ All of these findings suggest that GLP-1 mediates neuroprotective and neurotrophic actions. Interestingly, a triple GLP-1/GIP/glucagon receptor agonist was found to have neuroprotective effects on a transgenic mouse model of Alzheimer’s disease, as drug treatment increased neurogenesis and reversed memory deficit.^276^ Additionally, tri-agonist treatment resulted in apoptosis, synaptic loss, inflammation, amyloid levels and oxidative stress being reduced. How peripherally administered GLP-1 induces satiety mechanistically is unclear, but it is postulated that signals being generated by the binding of GLP-1 to its receptor on neurons in the GIT, hepatoportal bed and CNS mediate this effect.^32^,^107^,^234^ Interestingly, in contrast to this, a recent study concluded that central and peripheral GLP-1 systems suppress eating via independent gut-brain circuits in mice.^277^ The reported variation in the weight loss for the different GLP-1 analogues currently used to treat T2D is possibly related to their ability to penetrate the CNS, allowing for central GLP-1R binding.^197^,^200^ Given the controversy regarding the ability of GLP-1-based therapies in dementia treatment due to varying findings in multiple studies, especially in human studies, the future of these therapies to treat neurological disease is currently unclear.^32^ A recent study clearly demonstrated the therapeutic potential of GLP-1R agonists in dementia treatment, as T2D patients treated with these agonists had reduced incidence of dementia compared to placebo.^278^

##### Muscle, Adipose Tissue and Liver

Given the ability of GLP-1 to promote the incretin effect,^11^,^107^ this hormone has indirect actions on tissues such as skeletal muscle, adipose tissue and the liver through insulin- insulin acts as a “second messenger” of GLP-1. Therefore, GLP-1 indirectly promotes glucose uptake and storage in adipose tissue (excess glucose is stored as fat), skeletal muscle and cardiac muscle.^4^,^26^ Additionally, GLP-1, through the insulin secretion it induces, also promotes glycogen synthesis and inhibits gluconeogenesis in the liver, and also increases fat storage in adipocytes.^4^,^26^ Findings from certain studies suggest that GLP-1 has direct effects on the liver, adipose tissue and skeletal muscle and whether GLP-1R is expressed in these tissues is currently debated.^11^,^107^ GLP-1 was found to be able to bind to rat hepatocyte cell membranes, which resulted in increased inhibition of gluconeogenesis and enhanced glycogen synthesis after insulin release.^279^,^280^ One study found that GLP-1R is expressed in human hepatocytes obtained from liver biopsies, and treatment with GLP-1 analogues enhanced insulin sensitivity and fatty-acid catabolism, suggesting that these analogues could improve hepatic insulin resistance in patients with nonalcoholic fatty liver disease and nonalcoholic steatohepatitis.^281^ A more recent study found that GLP-1R expression was increased in hepatocytes from non-alcoholic steatohepatitis liver samples in comparison to healthy livers, implying that GLP-1R signalling plays a role in liver pathology.^282^ In strips of human skeletal muscle, GLP-1 promoted glycogen synthesis and inhibited glycogen phosphorylase activity- comparable findings were generated with L6 myotubes treated with GLP-1, as insulin-stimulated glycogen synthesis was increased.^283–285^ Interestingly, in mice, it was shown that GLP-1 secretion is increased during exercise and that overexpression of GLP-1 in mouse skeletal muscle enhanced endurance and glycogen synthesis.^286^ It has also been suggested that GLP-1 has synergistic effects on adipocytes with insulin, as basal and acute insulin-stimulated glucose uptake was enhanced by GLP-1 and exendin-4 in differentiated 3T3-L1 adipocytes.^287–289^ A study found that liraglutide therapy in mice decreased visceral fat, which is associated with metabolic diseases, and relatively increased subcutaneous fat, with lipogenesis being suppressed or enhanced, respectively. The expression of browning-related genes was upregulated in subcutaneous white adipose tissue in this study.^290^ These observations imply that GLP-1R signalling can redistribute body fat and promote browning remodelling in white adipose tissue, which may have clinical relevance. However, several studies found that GLP-1 did not enhance insulin actions in humans.^107^ Further, a study found that after T2D patients had bariatric surgery, GLP-1R expression in adipose tissue did not affect metabolic outcomes such as insulin sensitivity improvement, T2D remission and weight loss.^291^ Therefore, it is a matter of debate currently whether GLP-1 enhances the insulin actions on adipocytes, liver and skeletal muscle. If GLP-1 does enhance insulin actions and/or mediates direct effects in these tissues then the receptor(s)/mechanisms that mediate these effects are currently unclear.

##### Bone

It has been elucidated that GLP-1 also influences bone metabolism.^120^ GLP-1R-deficient mice exhibit cortical osteopenia, bone fragility, increased osteoclastic numbers and enhanced bone resorption activity.^292^ GLP-1 indirectly affects bone by upregulating calcitonin, which in turn inhibits bone resorption.^120^,^293^ However, one study reported that exendin-4 induced bone formation in rats.^294^ Whether GLP-1-based therapies have any beneficial effects on bone metabolism in humans is currently elusive/unclear.^295^ However, the large cardiovascular outcome trials of GLP-1R agonists have not found any obvious effects of these therapies on bone.^296–298^

##### Lung

Currently, it is thought that GLP-1 has no direct effects on the lungs, but this is an area of ongoing research.^11^,^107^ One study found that GLP-1 increased macromolecule secretion from neuroendocrine cells in the lungs.^299^ More recent studies have found that GLP-1R agonists can improve lung function in patients/animals with lung pathology and could be a new treatment for patients with lung diseases such as chronic obstructive pulmonary disease and asthma.^300^,^301^ However, the large cardiovascular outcome trials of GLP-1R agonists have not found any obvious effects of GLP-1 therapies on the lung.^295–297^ Further research is needed into the effects of GLP-1R agonists on lung function.

#### GLP-1R Knockout Mice

Studies investigating the effect of GLP-1R knockout/inactivation have generated some interesting observations. In 1996, GLP-1R knockout mice were first developed and it was found that they exhibit increased levels of blood glucose following oral glucose challenge in association with a reduction in levels of circulating insulin.^302^ However, studies have subsequently generated conflicting findings as to whether glucose tolerance is normal or impaired in these mice.^303^ Interestingly, intracerebroventricular administration of GLP-1 did not inhibit feeding in GLP-1R knockout mice but did in wild-type mice, suggesting that GLP-1R signalling mediates satiety, although no evidence for abnormal body weight or feeding behaviour was reported in GLP-1R knockout mice. In 2014, Ussher and colleagues inactivated GLP-1R in adult mouse cardiomyocytes and unexpectedly found that liraglutide administration after left anterior descending (LAD) artery occlusion induced increased heart rate and survival in these mice, although there was no difference in survival/adaptive responses or left ventricular remodelling following LAD coronary artery occlusion between GLP-1R inactivated and control mice that were not treated with any GLP-1R agonist.^304^ However, the basal 24h heart rate was significantly lower in GLP-1R inactivated mice. This implies that GLP-1R is required for the control of heart rate in mice in the absence of pathology, but endogenous cardiomyocyte GLP-1R activity is not required for both adaptive responses to ischemic or cardiomyopathic injury and GLP-1 analogue-induced cardioprotection or enhanced chronotropic activity.

**Figure 6:**
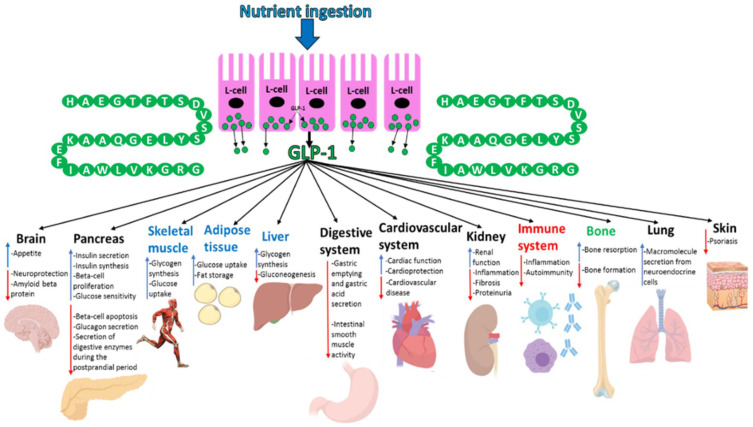
An overview of the effects/possible effects that GLP-1 has on various organs after it has been secreted into the circulation from L-cells of the gut in response to nutrient ingestion. Organs highlighted in blue are not known to express GLP-1R but GLP-1 has been demonstrated to mediate insulin-like effects on these tissues during experimental settings- it is, however, correct to state that GLP-1 does have indirect effects on these organs in humans due to its ability to promote the incretin effect. GLP-1R mRNA has been detected in immune cells and GLP-1 has been shown to regulate the immune system (highlighted in red) activity during experimental settings. GLP-1 has also been shown to influence bone (highlighted green) metabolism in rodents but the effects of GLP-1 on human bone are currently elusive. This figure and the information in its legend are adapted from these studies.^11^,^26^,^107^,^108^,^112^,^120^,^192^

Another study was able to successfully knock down GLP-1R expression in mice islet beta-cells by 70–80% which resulted in these mice having elevated fasting glucose plasma levels, but normal islet expression of insulin and proglucagon mRNA transcripts.^305^ Strikingly, GSIS and oral glucose tolerance were normal in these mice, and GLP-1 levels were not altered but interestingly GIP levels were decreased. Evidence was also provided from this study that GLP-1 controls glucose tolerance by extrapancreatic means, as GLP-1R antagonist administration increased plasma glucose levels in these mice during an oral glucose tolerance test. Intraperitoneal GLP-1 was shown to lower plasma glucose in GLP-1R knockdown mice without increasing insulin secretion, whereas intravenous GLP-1 did not affect glucose or insulin levels. Interestingly, one study found that GLP-1R knockout mice were protected from high-fat diet-induced insulin resistance and had enhanced insulin-stimulated skeletal muscle glucose uptake, and these mice also exhibited enhanced action of insulin on hepatocytes as well as decreased hepatic and skeletal muscle triglyceride accumulation.^306^ A recent study found that the insulin response to intravenous glucagon was preserved in GLP-1 receptor knockout mice but that glucagon’s insulinotropic actions were suppressed in isolated islets from GLP-1 receptor knockout mice.^121^ Additionally, in wild-type mice, the GLP-1 receptor antagonist substantially suppressed glucagon-induced insulin secretion. These data suggest that GLP-1 receptors contribute to the insulinotropic action of glucagon and that in GLP-1R knockout mice there is some extrapancreatic mechanism that compensates for the loss of glucagon-induced insulin secretion via GLP-1R. Interestingly, GLP-1R knockout mice were reported to exhibit similar or increased insulin secretion compared to wild-type mice in response to glucose loads, with glucose tolerance maintained in a recent study.^303^ From the observations in this study it seems that GLP-1R knockout mice adapt to the absence of GLP-1R, and are able to maintain glucose tolerance and desirable plasma insulin levels in response to glucose challenges. Figure 6 summarises the targets and actions of GLP-1.

In summary, GLP-1 and GLP-1R show expression in a variety of tissues and the best-studied and most currently clinically relevant effects of GLP-1 is its ability to act as an incretin hormone and reduce appetite, given the global prevalence of T2D and obesity.^26^,^32^ Even though the effects of GLP-1 on other tissues are not well characterised, it is becoming increasingly clear that this hormone does have physiological effects on a variety of tissues throughout the body, and a better understanding of the extrapancreatic effects of GLP-1 may have future therapeutic potential for diseases other than T2D.^11^

#### GIP

**Figure 7:**
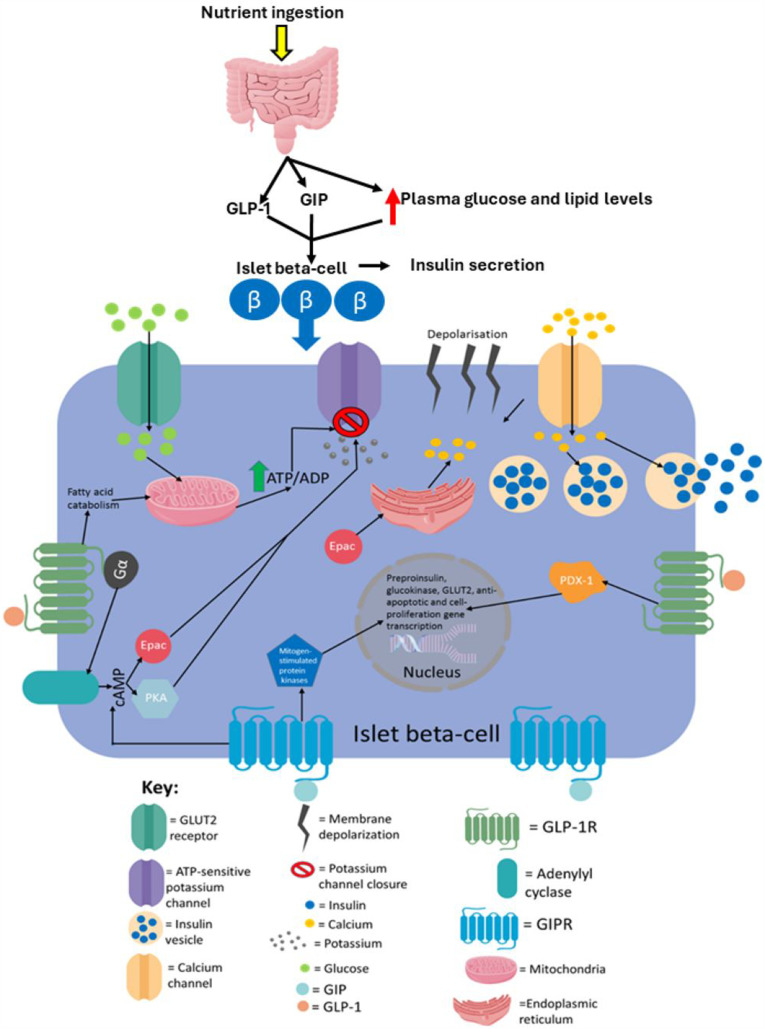
An overview of the processes in islet beta-cells that mediate incretin-induced insulin secretion. Upon entry into islet beta-cells from the circulation via GLUT2, glucose undergoes catabolism to raise ATP levels, which results in the closure of ATP-sensitive potassium channels, causing membrane depolarisation and subsequent calcium influx. The resulting calcium influx then triggers insulin secretion. The binding of GLP-1 and GIP, which are secreted from the intestine into the circulation in response to nutrient ingestion, to GLP-1R and GIPR, respectively, enhances glucose-stimulated insulin secretion by initiating cyclic adenosine monophosphate (cAMP) production via activation of adenylyl cyclase by Gα, which, in turn, activates protein kinase A (PKA) and exchange protein directly activated by cAMP (Epac). PKA and Epac further promote potassium channel closure which indirectly assists with extracellular calcium influx. Also, Epac directly induces calcium release from the endoplasmic reticulum, whereas PKA is thought to increase the permeability of calcium channels to enable a more rapid influx of calcium. The now elevated intracellular calcium levels further enhance the exocytosis of insulin vesicles. GLP-1R activation also induces transcription of the preproinsulin, glucokinase and GLUT2 genes allowing for further insulin production, glucose catabolism and glucose uptake, respectively, via activation of the PDX-1 transcription factor and its translocation to the nucleus. PDX-1 activation also induces transcription of genes involved in proliferation, neogenesis and apoptotic resistance. Additionally, GLP-1R activation promotes intracellular lipid catabolism which is thought to provide the mitochondria with more metabolic fuel to further raise the ATP/ADP ratio- ATP is needed for both phases of insulin secretion. This figure and the information in its legend are adapted from these studies.^11^,^55^,^107^,^112^,^120^,^192^

GIP is a 42aa-long hormone produced by K cells of the upper small intestine.^120^,^307^ The preproGIP gene, which is located on chromosome 17q21.3–q22, consists of 6 exons. After preproGIP (153aa long) is produced, it undergoes proteolytic processing to produce GIP.^120^ GIP levels, similar to GLP-1 levels, increase after nutrient ingestion: fasting levels in the blood range from 5–20pM and this increases to 50–100pM after glucose ingestion and 100–150pM after ingestion of mixed nutrients.^308^,^309^ Recently, experimental evidence has emerged that the nervous system may also regulate GIP secretion as galanin was shown to be able to inhibit both murine GLP-1 and GIP secretion by acting on its receptor expressed in L and K cells, respectively.^310^ GIP is not as potent as GLP-1 and has a longer half-life of ~5 minutes, but it is also inactivated by DPP-IV enzymes in circulation.^11^,^120^,^311^ GIP, to date, is thought to mediate its insulinotropic effects after binding to GIPR via similar intracellular pathways to GLP-1.^120^ GIPR activation results in ~42% of postprandial insulin secretion.^17^,^119^ GIPR knockout mice have impaired tolerance to oral glucose load but not to intraperitoneal glucose injection, confirming the importance of GIP in mice and its dependence on its receptor to mediate the incretin effect.^312^ Interestingly, GIP has also been shown to have anti-apoptotic and proliferative effects on islet beta-cells during in vivo and in vitro studies, as it has been shown to activate mitogen-stimulated protein kinase pathways.^120^ It has not been established if GIP actives PDX-1 similarly to GLP-1, but the current consensus in the literature is that GIP and GLP-1 activate both similar pathways and different pathways to promote their anti-apoptotic and proliferative effects.^120^,^210^ Figure 7 summarises how GIP and GLP-1 binding to their receptors promotes the incretin effect.

There are more prominent differences between these 2 hormones’ effects on islet alpha-cells: the effects of GLP-1 and GIP on glucagon secretion are opposite.^120^ GIP infusion was first shown to promote glucagon secretion in the 1970s, as this resulted in counteracting glucagon suppression induced by glucose in rats.^313^ Similar observations were obtained from healthy humans during euglycemia and T2D patients during meal-tolerance studies.^314^,^315^ Given GIP’s ability to promote glucagon secretion, it has been considered inappropriate to use clinically to treat T2D as it would amplify the hyperglycaemia associated with this disease.^120^ Additionally, islet beta-cells become irresponsive to GIP in T2D.^11^,^316^ Currently, there, therefore, seems a minimal clinical benefit of using GIP analogues in T2D treatment. Given GIP’s clear ability to act as the most important incretin hormone, it is surprising that it also augments glucagon secretion. It seems very unlikely that the glucagon released by GIPR activation counteracts the incretin effect, as this would result in a counterproductive postprandial response that would not promote metabolic homeostasis in the individual. There must be a currently unidentified physiological beneficial effect of GIP’s ability to promote glucagon release in response to postprandial nutrient loads. Interestingly, GIP has been shown to stimulate glucagon secretion even with high glucose in T2D patients, which implies that inappropriate GIP/GIPR activity contributes to the T2D disease phenotype.^317^ It has been shown that fatty acids strongly enhance GIP secretion, and this hormone then subsequently plays an important role in mediating fat uptake into adipose tissue.^318^,^319^

GIPR has been detected in adipose tissue and genetic ablation of GIPR in mice has clearly shown the importance that GIP has on fat uptake: GIPR-deficient mice did not develop weight gain and adiposity to the same degree as control mice even though they were on the same high-fat diets.^320^ Interestingly, the GIPR-deficient mice had increased oxygen consumption and respiratory quotient indicating that they used the excess fat to generate ATP.^321^,^322^ Further evidence that GIPR-deficient mice catabolised the excess fat is provided by the observation that these mice had enhanced adiponectin secretion, which induces fat oxidation in muscle and raises the respiratory quotient.^321^ Genetic ablation of GIPR in leptin-deficient obese (*ob/ob*) mice was shown to ameliorate insulin insensitivity and obesity by increasing energy expenditure without significantly affecting insulin secretion.^320^ These observations were confirmed in *ob/ob* mice and high-fat-fed mice when they were treated with a GIPR antagonist, and also in mice lacking GIP-secreting K cells, confirming the important role of GIP in fat accumulation.^323^,^324^ Although GIP is known to increase the activity of lipoprotein lipase, an enzyme that promotes the uptake of fats from the circulation into adipocytes for storage, it has not been well elucidated mechanistically how GIPR activation promotes this.^120^ In humans, higher serum fasting GIP concentrations were associated with an unhealthy body fat distribution independent of plasma insulin concentration, and genome-wide association studies found associations between body mass index, body fat distribution and increased visceral fat accumulation with single nucleotide polymorphisms of the GIPR locus.^325^ GIPR is downregulated in adipose tissue during insulin-resistant states, such as obesity, and several studies suggest that GIP augments insulin actions in adipose tissue.^326^,^327^ Interestingly, one study found that GIPs ability to augment insulin actions on adipose tissue was blunted in human adipocytes from obese but not lean individuals.^326^ Hence, loss of GIPR signalling may play a role in the pathogenesis of insulin resistance and the manifestation of obesity.

GIP also has important effects on bone formation.^120^ GIPR has been detected in bone tissue, and ovariectomy-induced bone loss was shown to be suppressed by GIP administration.^328^

**Figure 8:**
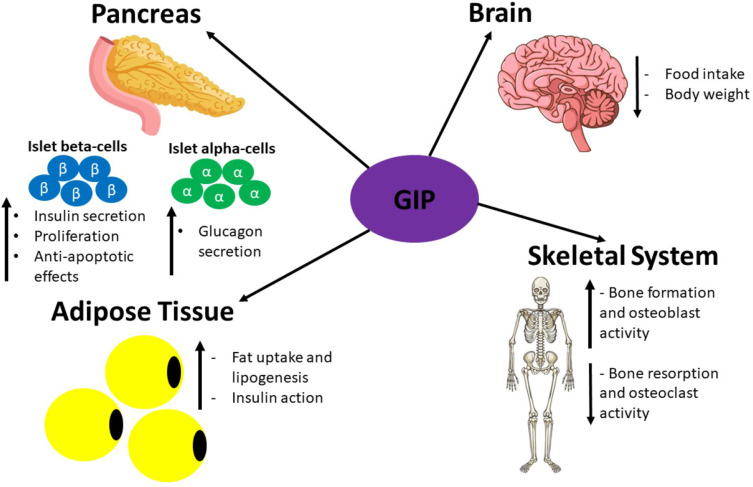
A summary of the direct effects that GIP has on various organs expressing GIPR upon its release during the postprandial period. This figure and the information in its legend are adapted from these studies.^119^,^120^,^312–315^,^318^,^319^,^321^,^325^,^329^,^334–338^

The importance of endogenous GIP was then demonstrated by the effect of GIPR deficiency in mice: these mice had thinner bone trabeculae resembling that observed in osteoporosis.^329^ The number of osteoclasts was increased in GIPR-deficient mice and in vitro studies have shown that GIP suppresses the apoptosis of osteoblasts.^120^ GIP transgenic mice have displayed enhanced bone formation mediated through the suppression of osteoclasts and prevention of osteoblast apoptosis by GIP.^330^ GIP may also promote calcium uptake into bone tissue from circulation as postprandial plasma calcium levels are increased in GIPR-deficient mice.^328^ Over recent years, studies have found that GIP treatment in humans reduces bone resorption, with one study also finding that this hormone additionally increases the rate of bone formation.^331–333^ A recent study also found that GIP reduced human osteoclast activity and osteoblast survival during in vitro experiments.^334^ Hence, GIPR activation-based therapies may have the potential for bone pathology treatment. Recent rodent studies have also generated evidence suggesting that GIP action through GIPR lowers body weight and food intake via central neuronal signalling, given that these effects were not observed in CNS-GIPR knockout mice.^335^,^336^ Further, the GLP-1/GIP dual-agonist tested in this study, MAR709, lost its superior efficacy on body weight and food intake over GLP-1 in the CNS-GIPR knockout mice, suggesting that this dual-agonist mediates part of its action via the CNS-GIPR. GIPR expression has been detected in several cell types in the human hypothalamus and studies show that GIPR signaling can enhance satiety.^337^,^338^ Figure 8 summarises the direct effects of GIP in tissues that express GIPR.

#### Double Incretin Receptor Knockout (DIRKO) Mice

DIRKO mice have normal body weight and do not have improved glycaemic response following exogenous administration of GIP or the GLP-1R agonist exendin-4.^339^ The hypoglycaemic response to exogenous insulin was normal in these mice. However, GSIS was decreased following oral but not intraperitoneal glucose administration in DIRKO compared to mice with just one of these receptors knocked out. This demonstrates how the incretin hormones are dependent on their receptor being present on islet beta-cells to mediate their insulinotropic effects. A subsequent study showed that single and dual incretin receptor knockout mice have impaired insulin secretion after high-fat feeding, but glucose control was only moderately altered due to maintained insulin sensitivity.^340^ Interestingly, DIRKO mice also exhibit increased energy expenditure which demonstrates that incretin hormone action may negatively regulate nutrient catabolism. Another study also found that DIRKO mice had reduced insulin secretion but enhanced insulin action and that these mice were protected from high-fat diet-induced obesity and insulin resistance further supporting the notion that incretin hormone action may negatively regulate nutrient catabolism.^341^ Interestingly, wild-type mice exhibited hyperglycemia and hyperinsulinemia in response to high-fat feeding but this was not observed in DIRKO mice.

In summary, both GIP and GLP-1 mediate the incretin effect but there is continuing emerging evidence that these hormones modulate physiological responses in extrapancreatic organs.^11^,^120^ Knocking out or inhibiting the action of one or both of the incretin receptors results in some expected and unexpected phenotypes.^120^ An unexpected phenotype produced by GIPR deficient *ob/ob* mice was that they lost weight by increasing their energy expenditure, which is surprising as their insulin levels were not significantly altered in comparison to control mice, and therefore their energy expenditure should have been similar to control mice.^340^

### Tri-Agonist for GLP-1R, GIPR and GCGR

Recently, a triple agonist for GLP-1R, GIPR and GCGR was developed and tested on diet-induced obese mice.^342^ The tri-agonist lowered body weight in diet-induced obese mice by 26.6% after 20 days of treatment, whereas the GLP-1R/GIPR coagonist lowered body weight by 15.7%, and it was found that the action of the tri-agonist occurred in a dose-dependent manner. Both the GLP-1R/GIP coagonist and the tri-agonist similarly reduced blood glucose levels and improved glucose tolerance without induction of hypoglycaemia, and this demonstrates that chronic GCGR agonism does not overpower the anti-hyperglycemic effects of GLP-1R and GIPR activation. Surprisingly, tri-agonist treatment lowered plasma insulin levels to a greater extent than the GLP-1R/GIP coagonist, which may indicate greater insulin sensitivity. Chronic GCGR activation should increase plasma glucose levels and insulin levels would then need to rise to promote normoglycaemia during tri-agonist treatment, but this is not the case. Interestingly, no difference in food intake was observed between wild-type mice treated with the dual incretin GLP-1/GIP coagonist and those treated with the tri-agonist despite the difference in weight loss, and this was found to be due to significantly higher ATP expenditure in tri-agonist-treated diet-induced obese mice. The potential of the tri-agonist and the dual incretin GLP-1/GIP coagonist to antagonise the manifestation of spontaneous diabetes was examined in mouse models of T2D.

The tri-agonist treatment prevented excessive weight gain in vehicle-treated mice to a greater degree than the GLP-1/GIP coagonist, and this observation was not due to any alterations in cumulative food intake. The observations here from both the tri-agonist and coagonist treatments are interesting, as numerous studies have indicated that GIPR activation results in weight gain, not weight loss.^343^ The tri-agonist also protected these mice from fasting hyperglycaemia to a greater extent than the coagonist. Interestingly, the tri-agonist also preserved the islet architecture by significantly reducing alpha-cell infiltration into the core of pancreatic islets. Glycaemic improvements were maintained in Zucker diabetic fatty rats 3 weeks after treatment cessation even though they had gained body weight and were similar in mass to vehicle-treated controls, demonstrating that the tri-agonist delays T2D progression in rodent models of spontaneous diabetes. This study also demonstrated that the effects of the tri-agonist are dependent on an excess of nutrient storage as weight and food intake were not altered in lean mice even after chronic treatment with the tri-agonist. Another recent study,^344^ which examined the impacts of another GLP-1, GIP and GCGR tri-agonist in rodents, found that treatment with this agonist decreased body weight, food intake and hyperglycaemia. Additionally, this agonist ameliorated diabetic nephropathy-related diseases in this study. These findings suggest that GLP-1, GIP and GCGR tri-agonists have the therapeutic potential to alleviate diabetic post-diagnosis complications and provide more efficacious treatments for patients. A recent study found that the triple agonist retatrutide for GLP-1R, GIPR and GCGR substantially induced weight loss in obese adults, demonstrating the clinical potential of this triple agonist for obesity treatment.^345^ Another triple agonist for these receptors, LY3437943, was found to induce robust reductions in plasma glucose and bodyweight in overweight individuals with T2D, demonstrating that this triple agonist could be a future therapeutic option for T2D treatment.^346^

### Future Perspectives

This review has attempted to summarise the complexity of the regulation of metabolic homeostasis by discussing the known/speculative actions of the incretin hormones and the metabolic hormones that are produced by the pancreatic islets. It is correct to state that many of the hormones involved in metabolism influence each other’s action by both indirect and direct mechanisms to promote metabolic homeostasis, as discussed in this review. The GLP-1R agonists appear a promising more efficacious pharmacological treatment option for obesity given the success of liraglutide and semaglutide, and hopefully in the future clinical trials will investigate the ability of the other relevant agonists to induce weight loss in obese individuals with/without diabetes.^239^ Studies have demonstrated that GLP-1 mediates several beneficial extrapancreatic actions on both tissues which either express GLP-1R or do not,^11^ and clinical trials have found that some GLP-1-mimetic-based therapies alleviate T2D-associated cardiovascular diseases.^242^ This suggests that GLP-1R agonists could alleviate the systemic extrapancreatic pathology associated with T2D,^347–349^ and have more clinical relevance than just improving insulin secretion in patients. Table 1 presents the GLP-1R agonists currently used in T2D treatment and their reported effects on disease-associated cardiovascular and kidney pathology in long-term human studies.

**Table 1:** Current GLP-1R agonists used in T2D treatment and their reported effects on cardiovascular and kidney pathology

GLP-1R Agonist Generic Name (Trade Name)	Half-Life/ Dosing/Route of Administration	Effects on Cardiovascular Pathology	Effect on Kidney Pathology	Study/Reference
Exenatide (Byetta)	2.4 hours/Twice daily/Injection	There has been no formal cardiovascular outcome trial (CVOT)	Not investigated	Best et al (2011)^350^
Lixisenatide (Lyxumia)	4 hours/Once daily/Injection	The CVOT for lixisenatide confirmed CV safety but no evidence of superiority concerning CV outcomes.	Not investigated	ELIXA^298^
Liraglutide (Victoza)	12 hours/Once daily/Injection	Superiority for CV outcomes versus placebo (composite of CV death, non-fatal myocardial infarction, non-fatal stroke) confirmed.	Not investigated	LEADER^351^
Dulaglutide (Trulicity)	90 hours/Once weekly/Injection	Superiority for CV outcomes versus placebo (composite of CV death, non-fatal myocardial infarction, non-fatal stroke) confirmed.	Reduced renal outcomes (first occurrence of new macroalbuminuria, a sustained decline in eGFR of 30% or more from baseline, or chronic renal replacement therapy)	REWIND^352^
Exenatide-LAR (Bydureon)	96 hours/Once weekly/Injection	The CVOT for exenatide LAR confirmed CV safety but no evidence of superiority concerning CV outcomes.	Not investigated	EXSCEL^296^
Semaglutide (Ozempic)	165–184 hours/Once weekly/Injection	CV safety confirmed in T2D; superiority for CV outcomes awaited from the definitve CVOT (SOUL). SELECT has confirmed superiority versus placebo for CV outcomes in people with obesity without diabetes.	FLOW study currently investigating	SUSTAIN-6^297^ and SELECT^247^
Semaglutide (Rybelsus)	165–184 hours/Once daily/Oral	CV safety confirmed in T2D; superiority for CV outcomes awaited from the definitive CVOT (SOUL).	Not investigated	PIONEER 6^353^

The conflicting observations between human and animal studies determining how various hormones’ actions regulate metabolic homeostasis, especially GLP-1, demonstrate that the findings from animal studies may have reduced clinical implications. Although this review has covered the activity of many important hormones involved in promoting metabolic homeostasis, many other molecules and hormones such as gastrin and noradrenaline are involved in this process,^40^ and their role in promoting metabolic homeostasis in healthy individuals and how their activity could become dysfunctional and promote pathology has not been discussed. Interestingly, GLP-1/GIP coagonists have also been reported to have weight and glucose-lowering effects in obese T2D patients despite numerous studies supporting the notion that GIP promotes weight gain/obesity.^343^ The additional unexpected observations from the GLP-1, GIP and GCGR tri-agonist studies^342^,^344^ warrant further investigation, as these studies highlight the incomplete understanding of metabolic hormonal synergism, and that further elucidation of this will likely lead to more efficacious treatments for diseases such as T2D. A better understanding of the complex biochemical interactions that regulate metabolic homeostasis in healthy individuals and the altered metabolic phenotype in patients with metabolic disorders will likely lead to the development of more efficacious treatments. The outcomes of the ongoing FLOW study will yield further insight into the potential of GLP-1 action-based therapies to alleviate pathology associated with obesity and T2D.^267^

## Conclusion

**Figure 9:**
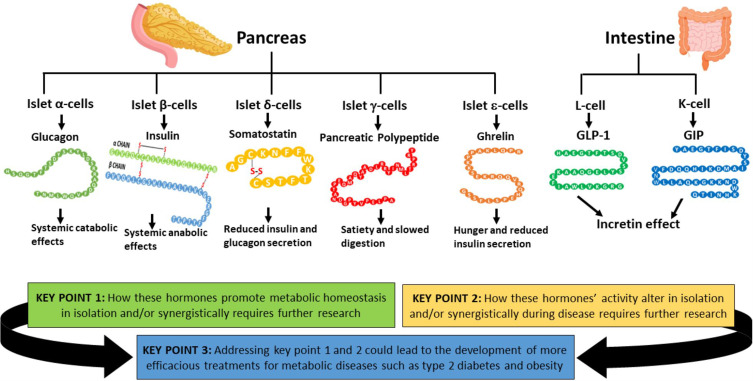
The hormones discussed in this review and the key points raised. The hormones involved in the regulation of metabolic homeostasis produced by the pancreas and the GIT discussed in this review are shown with their main/established effects. The key points of this review are displayed here, to highlight the potential importance of further understanding these hormones’ actions in isolation and synergistically during healthy and disease states could generate more efficacious and highly desirable treatments for metabolic disorders, such as T2D and obesity. This figure and the information in its legend are adapted from these studies.^26^,^32^,^42^,^48^,^107^,^120^,^144^,^149^,^165^,^166^

In conclusion, the regulation of metabolic homeostasis is a multifactorial, complex process, with the synergistic activity of the incretin effect and the pancreatic islets being integral for this being achieved. Further understanding of the complex mechanisms that regulate metabolic homeostasis and how these become dysregulated during metabolic diseases, which are currently a major global burden, will likely lead to the development of new highly desirable more effective therapeutic strategies to combat metabolic diseases such as T2D. Figure 9 shows the hormones and their main/established effects discussed in this review with the key points raised.

## Abbreviations

AA, amino acid; ADP/ATP, adenosine diphosphate/triphosphate; cAMP, cyclic adenosine monophosphate; CV, Cardiovascular; CNS, Central nervous system; DIRKO, Double incretin receptor knockout; DPP, Dipeptidyl peptidase; Epac, Exchange protein directly activated by cAMP; GIP, Gastric inhibitory peptide; GIPR, Gastric inhibitory peptide receptor; GIT, Gastrointestinal tract; GLP-1, Glucagon-like peptide-1; GLP-1R, GLP-1 receptor; GLUT, Glucose transporter; GPCR, G-protein coupled receptor; GSIS, Glucose-stimulated insulin secretion; GCGR, Glucagon receptor; IGF, Insulin-like growth factor; IGFR, Insulin-like growth factor receptor; LAD, Left anterior descending; PDX-1, Pancreatic duodenal homeobox-1; PKA, Protein kinase A; ROS, Reactive oxygen species; T1D/T2D, type 1/2 diabetes.
